# Methyl Substitution Destabilizes Alkyl Radicals

**DOI:** 10.1002/anie.202207477

**Published:** 2022-08-01

**Authors:** Eva Blokker, Willem‐Jan van Zeist, Xiaobo Sun, Jordi Poater, J. Martijn van der Schuur, Trevor A. Hamlin, F. Matthias Bickelhaupt

**Affiliations:** ^1^ Department of Theoretical Chemistry Amsterdam Institute of Molecular and Life Sciences (AIMMS) Amsterdam Center for Multiscale Modeling (ACMM) Vrije Universiteit Amsterdam De Boelelaan 1083 1081 HV Amsterdam The Netherlands; ^2^ Departament de Química Inorgànica i Orgànica & IQTCUB Universitat de Barcelona Martí i Franquès 1–11 08028 Barcelona Spain; ^3^ ICREA Pg. Lluís Companys 23 08010 Barcelona Spain; ^4^ Polymer Specialties, Nouryon Zutphenseweg 10 7418 AJ Deventer The Netherlands; ^5^ Institute of Molecules and Materials Radboud University Heyendaalseweg 135 6525 AJ Nijmegen The Netherlands

**Keywords:** Bond Dissociation Energy, Bonding Theory, Density Functional Calculations, Radicals, Substituent Effects

## Abstract

We have quantum chemically investigated how methyl substituents affect the stability of alkyl radicals Me_m_H_3−m_C⋅ and the corresponding Me_m_H_3−m_C−X bonds (X = H, CH_3_, OH; m = 0 – 3) using density functional theory at M06‐2X/TZ2P. The state‐of‐the‐art in physical organic chemistry is that alkyl radicals are stabilized upon an increase in their degree of substitution from methyl<primary<secondary<tertiary, and that this is the underlying cause for the decrease in C−H bond strength along this series. Here, we provide evidence that falsifies this model and show that, on the contrary, the Me_m_H_3−m_C⋅ radical is *destabilized* with increasing substitution. The reason that the corresponding C−H bond nevertheless becomes weaker is that substitution destabilizes the sterically more congested Me_m_H_3−m_C−H molecule even more.

## Graphical abstract

Radical stability *decreases*, *and not increases*, from methyl to primary to secondary to tertiary. This is not conflicting with the fact that the C−H bond becomes weaker along the corresponding series H_3_C−H, MeH_2_C−H, Me_2_HC−H, and Me_3_C−H. The resolution to this seeming contradiction is that the parent Me_m_H_3−m_C−H is *destabilized even more* upon methyl substitution than the radical Me_m_H_3−m_C⋅.

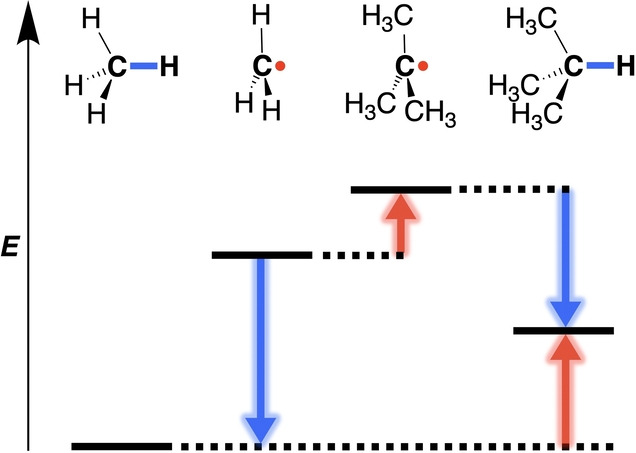

The C−H bond strength in simple alkanes decreases as the degree of substitution on the carbon atom increases, for example, along the series of methane (H_3_C−H), ethane (MeH_2_C−H), propane (Me_2_HC−H), and 2‐methylpropane (Me_3_C−H). The current explanation for this trend in C−H bond strength is that the alkyl radicals, formed from homolytic C−H bond dissociation, are stabilized by alkyl substitution and that this stabilization is enhanced as the number of stabilizing substituents increases.[^1^, ^2^, ^3^, ^4^] Radical stability is commonly quantified using the concept of “radical stabilization energy” (RSE). For the radical Me_m_H_3−m_C⋅ (m = 0 – 3), the RSE is defined through the isodesmic reaction in Equation (1) which relates its stability to that of the unsubstituted methyl radical as a reference system.[^5^, ^6^]

(1) $$\mathrm{Me}{}_{m}H{}_{3-m}C{}^{•}\;+\;H{}_{3}C-H\;\to \mathrm{Me}{}_{m}H{}_{3-m}C-H\;+\;H{}_{3}C{}^{•}\;\Delta H=\mathrm{RSE}$$

Experimental RSE values are 3.8 ± 0.5 kcal mol^−1^, 6.3 ± 0.5 kcal mol^−1^ and 8.4 ± 0.5 kcal mol^−1^ for primary, secondary and tertiary radicals, respectively, which are interpreted as indicating the larger stability of the substituted relative to the unsubstituted CH_3_⋅ radical.^[7]^

However, several authors have previously noted complications with this definition.[^5^, ^8^, ^9^, ^10^, ^11^, ^12^, ^13^, ^14^] For example, in the case of different bonds than C−H, the *trend* in RSE may change. A case in point is the C−O bond, which becomes stronger, not weaker, as the degree of substitution increases along the series methanol (H_3_C−OH), ethanol (MeH_2_C−OH), 2‐propanol (Me_2_HC−OH), and 2‐methyl‐2‐propanol (Me_3_C−OH).^[7]^ Thus, in this series of C−O bonds, the unsubstituted methyl radical emerges as the most stable radical, instead of the least stable, suggesting that the substituents would destabilize the radical center. This leaves us with the conflicting picture that RSE trends for C−H and C−O bonds suggest opposite behavior of the methyl groups on the stability of the radical.

Herein, we reveal the origin of the conflicting pictures suggested by the trends in RSE values as defined in Equation (1). And, more importantly, we show that methyl substituents, in fact, *destabilize* alkyl radicals in all cases studied. Whether the C−X bond in Me_m_H_3−m_C−X becomes weaker or stronger upon methyl substitution, depends on if the substituents destabilize the parent molecule more or less, respectively, than the corresponding radical Me_m_H_3−m_C⋅. To achieve our objectives, we have analyzed the carbon–substituent interaction in *both*, the parent molecule and the radical species, as shown in Scheme 1, for representative model systems X = H, CH_3_, and OH, using Voronoi Deformation Density (VDD) charges and Kohn–Sham molecular orbital (MO) theory at M06‐2X/TZ2P, as implemented in the ADF program.[^15^, ^16^, ^17^, ^18^, ^19^, ^20^]

**Scheme 1:**
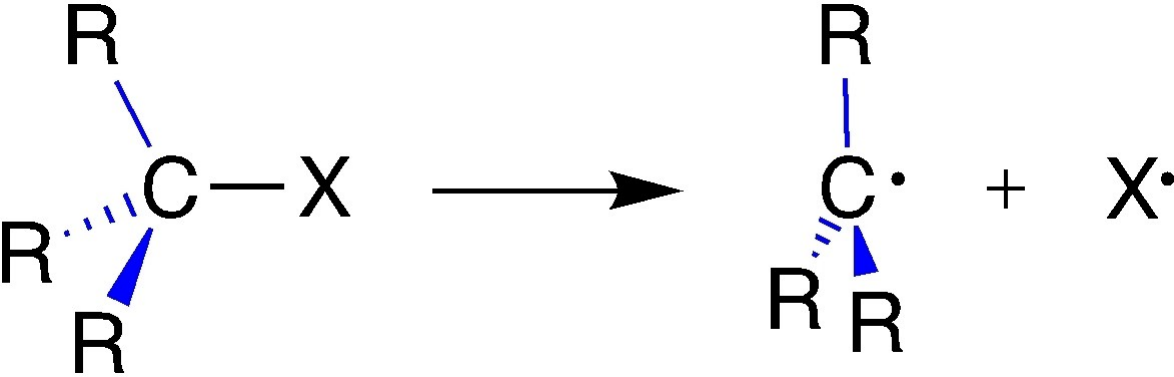
Interaction of the substituents R with the C−X moiety (left side) or with the radical center at C (right side) upon homolytic C−X bond dissociation.

Table 1 shows our computed Me_m_H_3−m_C−X bond dissociation enthalpies Δ*H*_BDE_ (BDEs) and bond lengths *d*_C−X_ for X = H, CH_3_, and OH. The C−H bond indeed weakens as the degree of methyl substitution increases, from 103.7 kcal mol^−1^ for H_3_C−H to 95.2 kcal mol^−1^ for Me_3_C−H.[^21^, ^22^] Likewise, the C−C bond also weakens as the degree of methyl substitution increases, although to a lesser extent, from 90.0 kcal mol^−1^ for H_3_C−CH_3_ to 86.3 kcal mol^−1^ for Me_3_C−CH_3_. At variance, the C−O bond does not weaken but becomes stronger as the degree of methyl substitution increases, namely, from 92.8 kcal mol^−1^ for H_3_C−OH to 95.8 kcal mol^−1^ for Me_3_C−OH. All C−X bonds become slightly longer upon increasing methyl substitution, up to ca. one hundredth of an Ångstrom, if one goes from m = 0 to m = 3 (Table 1).^[23]^

**Table 1:** Me_m_H_3−m_C−X (m = 0 – 3) bond dissociation energies and enthalpies (Δ*E*_BDE_, Δ*H*_BDE_), decomposition of Δ*H*_BDE_ using the thermochemical cycle in Scheme 2 [in kcal mol^−1^], and C−X bond lengths [in Å].^[a]^

Me_m_H_3−m_C−X	m	Name	Δ*E*_BDE_	Δ*H*_BDE_	Δ*H*_Par_(X,m)	Δ*H*_Rad_(m)	*d*_C−X_
H_3_C−H	0	methane	111.6	103.7	−331.9	−399.5	1.087
MeH_2_C−H	1	ethane	107.7	99.8	−318.3	−389.7	1.089
Me_2_HC−H	2	propane	104.7	96.9	−307.0	−381.3	1.091
Me_3_C−H	3	2‐methylpropane	102.7	95.2	−297.4	−373.5	1.093
							
H_3_C−CH_3_	0	ethane	97.4	90.0	−325.2	−399.5	1.525
MeH_2_C−CH_3_	1	propane	95.4	88.5	−313.9	−389.7	1.524
Me_2_HC−CH_3_	2	2‐methylpropane	93.8	87.3	−304.3	−381.3	1.526
Me_3_C−CH_3_	3	2,2‐dimethylpropane	92.3	86.3	−295.5	−373.5	1.529
							
H_3_C−OH	0	methanol	99.3	92.8	−337.8	−399.5	1.414
MeH_2_C−OH	1	ethanol	100.1	94.2	−329.5	−389.7	1.419
Me_2_HC−OH	2	2‐propanol	100.7	95.3	−322.2	−381.3	1.423
Me_3_C−OH	3	2‐methyl‐2‐propanol	100.6	95.8	−314.8	−373.5	1.428

[a] Computed at M06‐2X/TZ2P (298.15 K and 1 atm). See also Figure 1. Δ*H*_BDE_ of C^…^−H, C^…^−CH_3_, and C^…^−OH is 171.3, 164.3, and 154.5 kcal mol^−1^, respectively.

The C−X bond to a methyl group is weaker than the C−X bond to a hydrogen atom; for instance, H_3_C−CH_3_ has a BDE of 90.0 kcal mol^−1^, whereas H_3_C−H has a BDE of 103.7 kcal mol^−1^ (Table 1).^[23]^ This is also true for the C−X bond in the alkyl radicals Me_m_H_2−m_C⋅−X (Table S4), where the C−X bond for X = CH_3_ is weaker than the C−X bond for X=H, as well as for the unsubstituted carbon where C^…^−CH_3_ has a BDE of 164.3 kcal mol^−1^ and C^…^−H of 171.3 kcal mol^−1^ (see caption Table 1). This already shows that substituting a hydrogen atom for a methyl group gives a weaker bond, and thus destabilizes the species. As we have stated before, whether the C−X bond in Me_m_H_3−m_C−X becomes weaker or stronger upon methyl substitution, depends on if the methyl substituents destabilize the parent molecule more or less, respectively, than the corresponding radical Me_m_H_3−m_C⋅. To analyze how the homolytic C−X bond dissociation enthalpy Δ*H*_BDE_ depends on both, the bonding of substituents in the radical Me_m_H_3−m_C⋅ and in its parent Me_m_H_3−m_C−X, we have decomposed it into three terms, i.e., Δ*H*_Par_(X,m), Δ*H*_BDE_(C^…^−X), and Δ*H*_Rad_(m), associated with the three partial reactions of the thermochemical cycle shown in Scheme 2 (data in Table 1).[^24^, ^25^, ^26^]

**Scheme 2:**
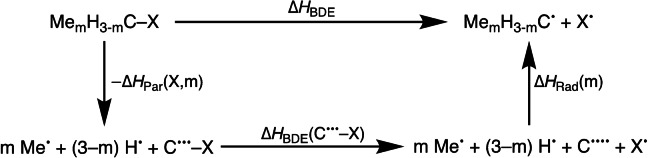
Thermodynamic cycle for the Me_m_H_3−m_C−X bond dissociation energy.

Δ*H*_Par_(X,m) is the overall bond enthalpy as the three separate substituents, that is, m Me⋅+(3−m) H⋅ for m = 0 – 3, combine with C^…^−X to form the parent molecule Me_m_H_3−m_C−X. Δ*H*_BDE_(C^…^−X) is the C−X bond dissociation enthalpy of the completely unsubstituted C^…^−X species, which is in the valence configuration of the CX moiety in Me_m_H_3−m_C−X. Δ*H*_Rad_(m) is the overall bond enthalpy as the three separate substituents mentioned above combine with C^….^ to form the radical Me_m_H_3−m_C⋅. Thus, we have the relationship of Equation 2:

(2) $$\Delta H{}_{\mathrm{BDE}}\;=\;\Delta H{}_{\mathrm{BDE}}\left(C-X{}^{•}{}^{•}{}^{•}\right)\;+\;\Delta H{}_{\mathrm{Rad}}\left(m\right)\;-\;\Delta H{}_{\mathrm{Par}}\left(X,m\right)$$

Note that Δ*H*_Rad_(m) is independent of X, whereas Δ*H*_BDE_(C^…^−X) is independent of the methyl and hydrogen substituents.

Δ*H*_BDE_ is thus determined by the intrinsic C−X bond dissociation enthalpy Δ*H*_BDE_(C^…^−X) of the unsubstituted C^…^−X species plus the *difference* in stabilization by the substituents of the radical, Δ*H*_Rad_(m), and the stabilization of the parent molecule by the same substituents, Δ*H*_Par_(X,m). Therefore, when altering the number of methyl substituents from 0 to m, the BDE of Me_m_H_3−m_C−X does not only depend on the change in stabilization of the radical,

(3) $$\Delta \Delta H{}_{\mathrm{Rad}}\left(m\right)\;=\;\Delta H{}_{\mathrm{Rad}}\left(m\right)\;-\;\Delta H{}_{\mathrm{Rad}}\left(0\right),$$

but also on the change in stabilization of the parent,

(4) $$\Delta \Delta H{}_{\mathrm{Par}}\left(X,m\right)\;=\;\Delta H{}_{\mathrm{Par}}\left(X,m\right)\;-\;\Delta H{}_{\mathrm{Par}}\left(X,0\right).$$

Consequently, the trend in Δ*H*_BDE_ upon increasing methyl substitution is determined by the *difference* between the two values, ΔΔ*H*_Rad_(m) and ΔΔ*H*_Par_(X,m). This insight is the key to understanding the origin of the substituent effects on the BDE of the Me_m_H_3−m_C−X bond.

The change in stabilization by the substituents in Me_m_H_3−m_C⋅ and in Me_m_H_3−m_C−X as a function of the number of methyl groups ΔΔ*H*_Rad_(m) and ΔΔ*H*_Par_(X,m), according to Equations (3) and (4), respectively, is plotted in Figure 1 and numerically displayed in Table 1 (similar destabilization is found with a variety of other density functionals; see Figure S1 and Table S2). The radical stability decreases from methyl to primary to secondary to tertiary, at odds with textbook knowledge.[^1^, ^2^, ^3^, ^4^] Substituting a hydrogen atom for a methyl group (Δm = 1) always effectively destabilizes both, the radical and the parent molecule. We can now explain the observed trends in C−X bond strength for X = H, CH_3_, and OH. The reason why the C−H bond weakens upon increased methyl substitution (Table 1) is that the radical Me_m_H_3−m_C⋅ is *destabilized less* than the corresponding parent Me_m_H_3−m_C−H is, along this series from m = 0 to 3 (Figure 1). Furthermore, the ΔΔ*H*_Par_(CH_3_,m) line increases less steeply from m = 0 to 3 than the ΔΔ*H*_Par_(H,m) line (Figure 1). Therefore, the C−C bond weakens, but less so than the C−H bond, namely, from 90.0 to 86.3 kcal mol^−1^ (Table 1). Lastly, the ΔΔ*H*_Par_(OH,m) line is below the ΔΔ*H*_Rad_(m) line (Figure 1). The radical is now *destabilized more* than the parent alcohol from m = 0 to m = 3, and this results in the C−O bond strengthening from 92.8 to 95.8 kcal mol^−1^ (Table 1).

**Figure 1:**
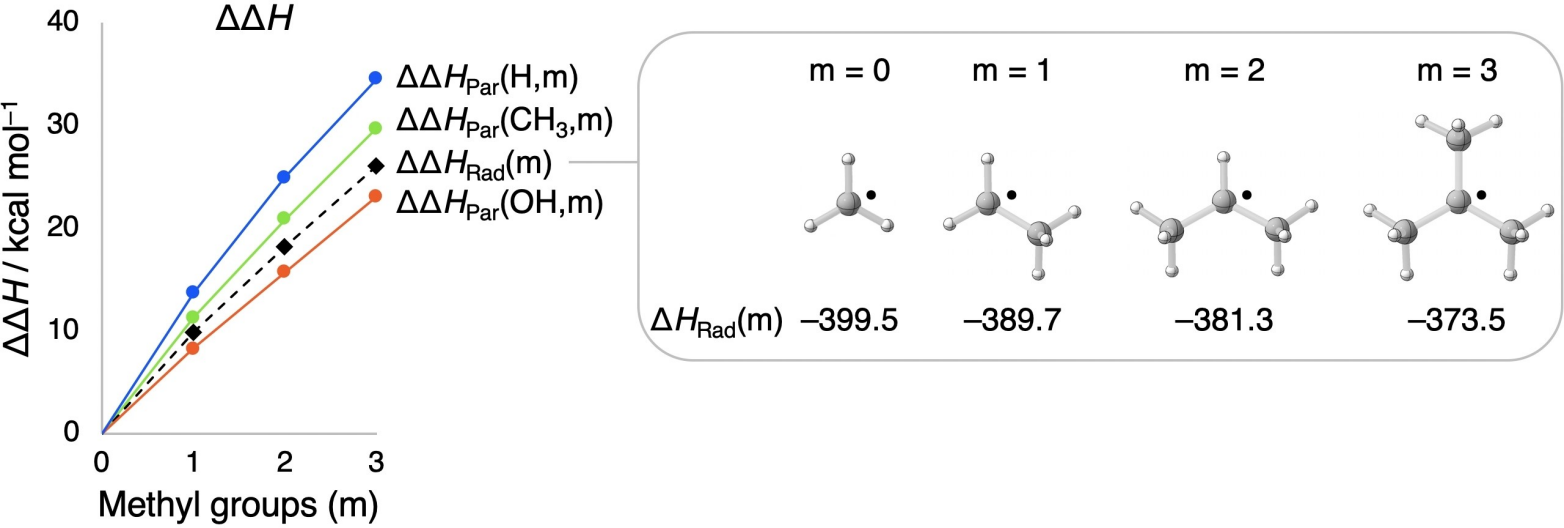
Effect (in kcal mol^−1^) of substituting hydrogens for m = 0 – 3 methyl groups on Δ*H*_Par_(X,m) and Δ*H*_Rad_(m) in Scheme 2 for X = H, CH_3_ and OH. Computed at M06‐2X/TZ2P at 298.15 K and 1 atm.

Next, we address the question *why* the C−O bond strengthens upon methyl substitution whereas the other C−X bonds weaken (Table 1). To this end, we have analyzed the features in the bonding mechanism that determine the trends in ΔΔ*E* for the two series of model systems with the most prominent difference in trend: those involving C−H bonds (weakening upon methyl substitution) and those involving C−O bonds (strengthening upon methyl substitution). Note that the trend in ΔΔ*E* determines in all cases the trend in ΔΔ*H* (compare Figures 1 and S2). Thus, in the following, we analyze ΔΔ*E*_Par_(X,m) and ΔΔ*E*_Rad_(m) and decompose these difference energies, associated with methyl substitution, into the corresponding difference in strain ΔΔ*E*_strain_ and the difference in interaction ΔΔ*E*_int_ (Figure 2).^[17]^ We recall that the interacting fragments to which Δ*E*_Par_(X,m) and Δ*E*_Rad_(m) refer, are Me_m_H_3−m_^…^ + CX^…^ and Me_m_H_3−m_^…^ + C^….^, respectively (see Scheme 2). For a given m, the bonding analysis is carried out at equal substituent–carbon distances (i.e. equal R−C distance from the fragment Me_m_H_3−m_^…^ to CX^…^ or to C^….^, with otherwise optimized geometry parameters), namely those based on the geometry of the corresponding parent with X = H, that is, Me_m_H_3−m_C−H. This approach prevents that a comparison of energy terms is skewed by geometrical relaxation effects.

**Figure 2:**
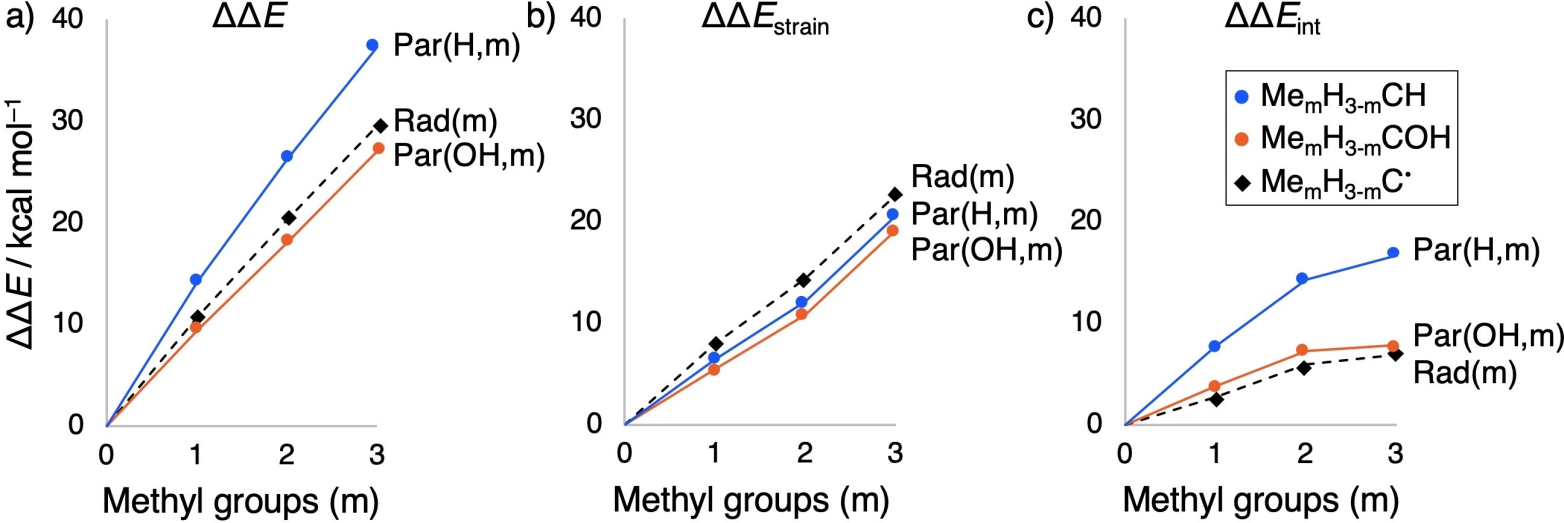
Effect (in kcal mol^−1^) of substituting hydrogens for m = 0 – 3 methyl groups on Δ*E*_Par_(X,m) and Δ*E*_Rad_(m) in Scheme 2, and their corresponding activation strain analysis, for X = H and OH. Computed at M06‐2X/TZ2P and, for each m, at equal substituent–carbon distances based on the geometry of Me_m_H_3−m_C−H.

In all cases, adding a methyl group (Δm = +1) destabilizes the system by both, a more destabilizing strain (ΔΔ*E*_strain_ > 0) and a less stabilizing interaction energy (ΔΔ*E*_int_ > 0; see Figure 2). This results from more steric (Pauli) repulsion in the following way. The larger methyl groups have more repulsion with the CX^…^ or C^….^ center and also have more mutual repulsion than the smaller hydrogen atoms. The enhanced Pauli repulsion upon methyl substitution shows up as an increasingly destabilizing term of the interaction energy (ΔΔ*E*_Pauli_ > 0 and ΔΔ*E*_int_ > 0; see Figures 2c and especially 3). But part of the enhanced Pauli repulsion is absorbed into an increasingly destabilizing strain (ΔΔ*E*_strain_ > 0; see Figure 2b) which is associated with a geometrical relaxation predominantly induced by the repulsion between the methyl substituents (*vide supra*).[^23^, ^27^, ^28^]

The reason that the C−H bond nevertheless becomes weaker for methyl substitution is that the parent Me_m_H_3−m_C−H suffers from a stronger increase in Pauli repulsion than the radical Me_m_H_3−m_C⋅ (Figures 2c and 3). Therefore, the ΔΔ*E*_int,Par_(H,m) line increases more steeply from m = 0 to 3 than the ΔΔ*E*_int,Rad_(m) line. The underlying cause is that the parent is sterically more crowded due to having a higher coordination number (i.e., 4) at the central carbon atom than the radical (i.e., 3). On the other hand, the reason that the C−O bond becomes stronger in the parent Me_m_H_3−m_C−OH is that the interaction with the methyl groups benefits from the presence of the OH‐group. Therefore, the ΔΔ*E*_Par_(OH,m) line increases less steeply from m = 0 to 3 than the ΔΔ*E*_Par_(H,m) line (Figure 2c). This is caused by a more stabilizing electrostatic interaction ΔΔ*V*_elstat_ (and thus interaction energy ΔΔ*E*_int_) with the methyl groups in Me_m_H_3−m_COH than in Me_m_H_3−m_CH (Figures 2c and 3), which is in line with the reduced electron density on the carbon atom in COH^…^ compared to CH^…^, as inferred from VDD analysis (Table S7). The same trends are obtained if the bonding analyses are computed at equal substituent–carbon distances stemming from the geometry of the Me_m_H_3−m_C⋅ radical (Figures S4 and S5).

**Figure 3:**
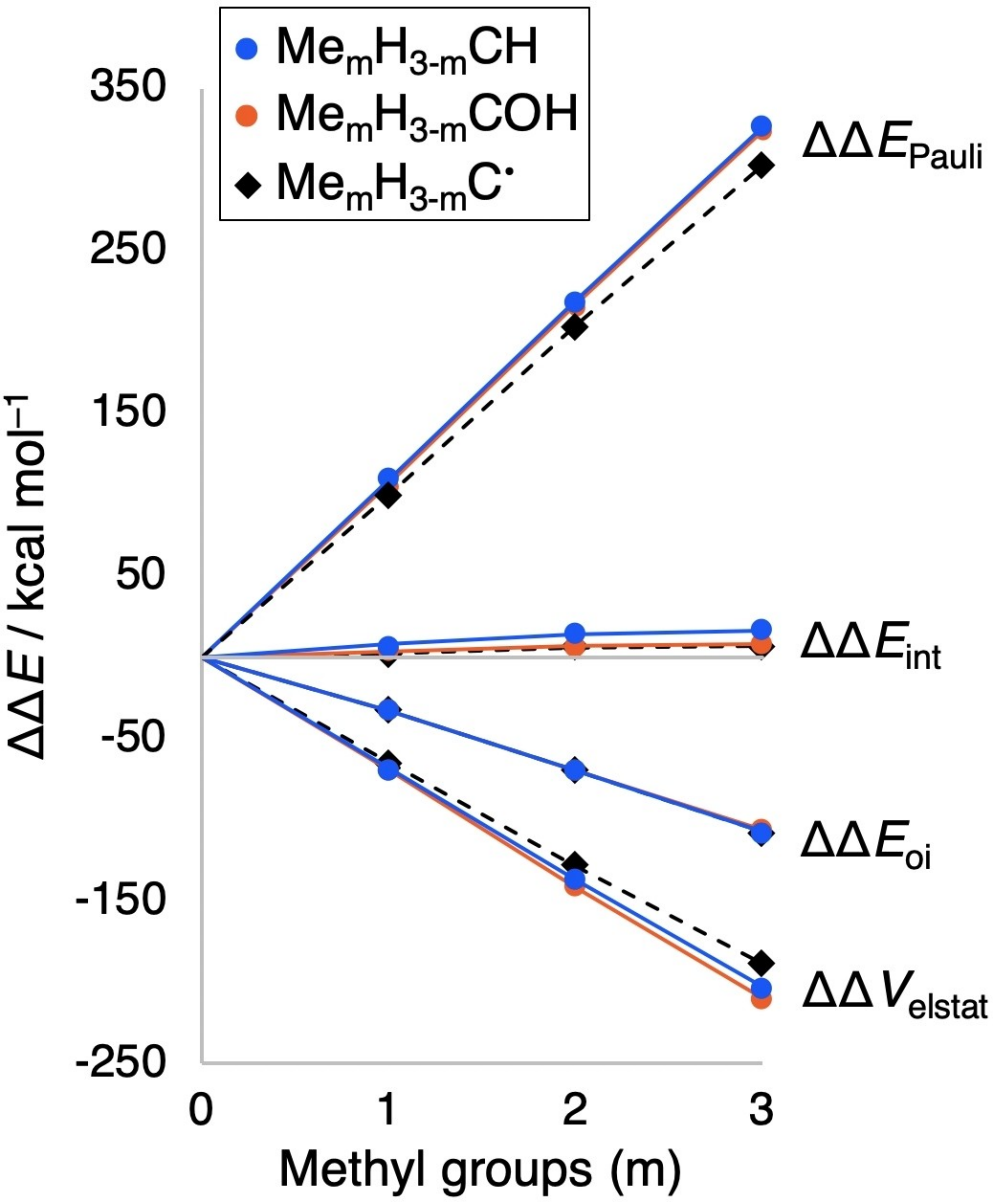
Effect (in kcal mol^−1^) of substituting hydrogens for m = 0 – 3 methyl groups on the energy decomposition analysis of Δ*E*_Par_(X,m) and Δ*E*_Rad_(m) in Scheme 2 for X = H and OH. Computed at M06‐2X/TZ2P and, for each m, at equal substituent–carbon distances based on the geometry of Me_m_H_3−m_C−H.

Finally, we address the role of hyperconjugation which, in the current literature, is invoked to explain why methyl substitution would stabilize the organic radical Me_m_H_3−m_C⋅.[^2^, ^3^] In the first place, we recall that we just showed that methyl substitution does *not* stabilize the organic radical. But does hyperconjugation occur at all? And if so, what is its effect?

Our MO analyses show that hyperconjugation occurs in both, the parent Me_m_H_3−m_C−H as well as the radical Me_m_H_3−m_C⋅, not only in the latter. In Figure 4, the important orbital interactions of A symmetry are shown, i.e., A_1_′ and A_2_′′ symmetry for the *D*_3h_‐symmetric methyl radical and A_1_ symmetry for the C_3v_‐symmetric tert‐butyl radical and parent molecules. We do find the textbook hyperconjugation which arises from the donor–acceptor interaction between occupied σ_C‐H_ orbitals on the methyl substituents and the radical p‐orbital at the central carbon atom, shown in blue in Figure 4b, but also an analogous donor–acceptor orbital interaction in the parent molecule, shown in Figure 4d. Note that, in the case of Me_m_H_3−m_C⋅, the antibonding combination of this hyperconjugative interaction constitutes the SOMO of the final organic radical (Figure 4b, blue), whereas in the parent Me_m_H_3−m_C−H, the corresponding orbital is closed‐shell (Figure 4d, blue). Our analyses reveal also other stabilizing 2‐center–3‐electron (2c–3e^−^) interactions and are shown in red for both the parent and the radical (Figure 4). And in fact, the largest contribution of additional stabilization from orbital interactions upon methyl substitution does not arise in A symmetry (in which the type of hyperconjugation occurs that is described in textbooks; see Figure 4, blue), but in E symmetry (see Figures S7 and S8). For instance, the orbital interaction stabilization from H_3_C⋅ to Me_3_C⋅ is in total −107.2 of which −39.0 kcal mol^−1^ comes from A symmetry and −82.4 kcal mol^−1^ from E symmetry (Table S8).^[17]^ Again, also in E symmetry, stabilizing hyperconjugative interactions occur in both the radical and the parent. Natural bond orbital (NBO) analyses also confirm that hyperconjugation occurs in both, the parent and the radical (Figures S10 and S11).

**Figure 4:**
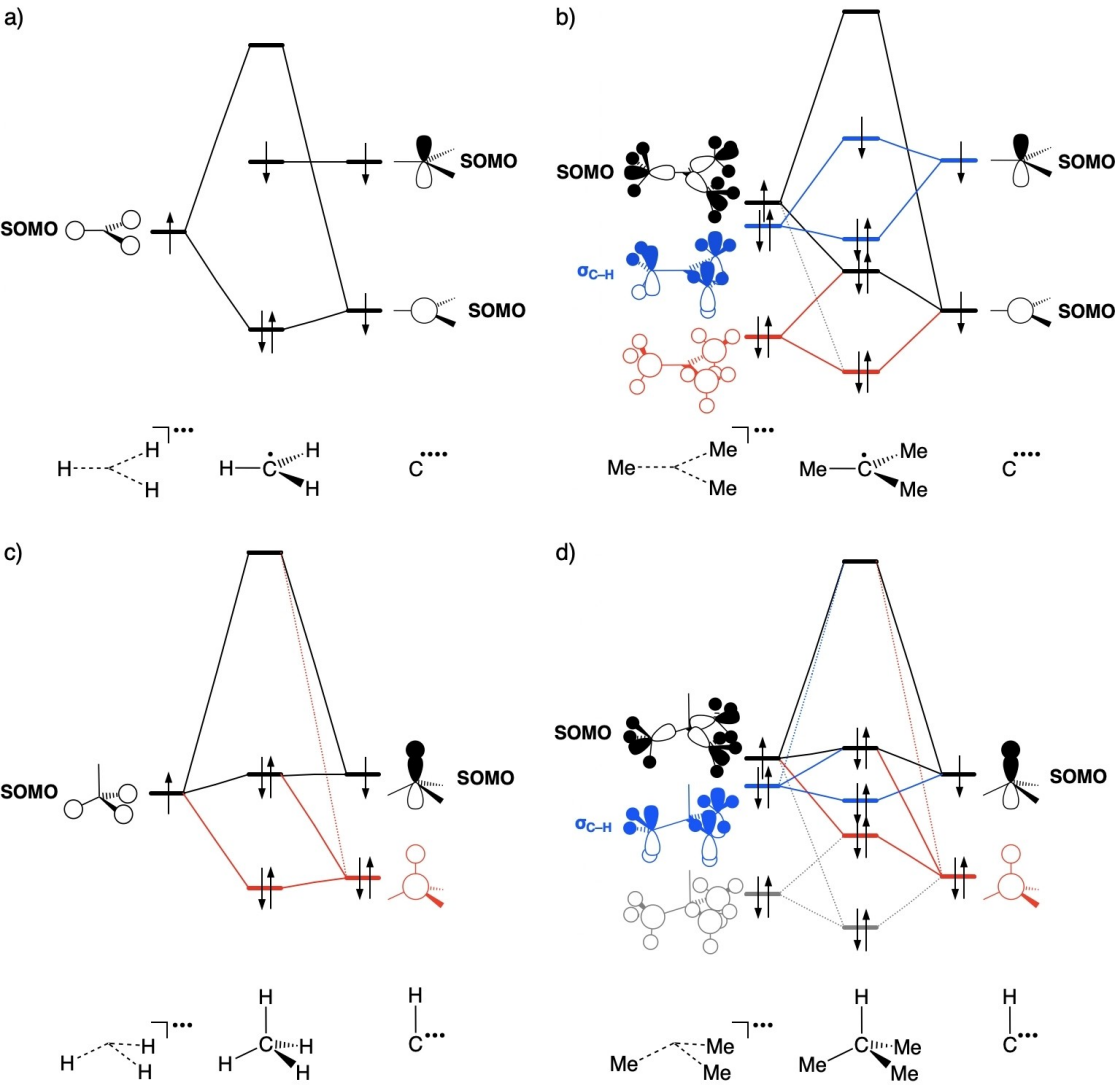
Schematic MO diagram, emerging from our KS‐MO analyses at M06‐2X/TZ2P, in A_1_′ and A_2_′′ symmetry for a) H_3_C⋅, and in A_1_ symmetry for b) Me_3_C⋅, c) H_3_CH and d) Me_3_CH. Interactions: 2c–2e^−^ in black, 2c–3e^−^ in red, 2c–3e^−^ hyperconjugation between Me_3_^…^ σ_C−H_ and C^….^ p SOMO or CH^…^ p‐type SOMO in blue.

Thus, the MO bonding analyses suggest that hyperconjugation has no, or no significant, effect on the relative stability of radicals and parent molecules. This picture is fully confirmed by the energy decomposition analyses (EDA), as can be seen from the virtually overlapping ΔΔ*E*_oi_ lines for radical and parents (Figure 3). Thus, the additional stabilization by orbital interactions upon introducing a methyl group (Δm = +1) is nearly exactly the same for the radical and the parent molecules.

In conclusion, our quantum chemical analyses reveal that, in contrast to common textbook knowledge, methyl substitution *destabilizes* organic radicals Me_m_H_3−m_C⋅ instead of making them more stable. The reason is disarmingly simple: the bond to a methyl group is less stable than the bond to a hydrogen atom, and there is more mutual repulsion between the larger methyl groups. Still, the C−H and C−C bond for Me_m_H_3−m_C−X (X=H, CH_3_) becomes weaker upon methyl substitution because the sterically more congested parent molecules (coordination number of central carbon is 4) are destabilized even more by methyl substitution than the radicals (coordination number of central carbon is only 3). Intriguingly, hyperconjugation has no significant effect on the relative stability of the radical and parent molecule. The current concept that methyl substitution stabilizes organic radicals is the consequence of a misinterpretation of the radical stabilization energies (RSE) which do not only depend on the stability of the radical but also on that of the parent molecule.

## Conflict of interest

The authors declare no conflict of interest.

## Supporting information

As a service to our authors and readers, this journal provides supporting information supplied by the authors. Such materials are peer reviewed and may be re‐organized for online delivery, but are not copy‐edited or typeset. Technical support issues arising from supporting information (other than missing files) should be addressed to the authors.

Supporting Information

## Data Availability

The data that support the findings of this study are available in the Supporting Information of this article.
