# Supplementary material for: Methyl Substitution Destabilizes Alkyl Radicals
Source: Angew Chem Int Ed Engl. 2022 Aug 1;61(36):e202207477. doi: 10.1002/anie.202207477 (PMC9545886; doi:10.1002/anie.202207477)
Supplement: Supplementary file 1 — Supporting Information [file ANIE-61-0-s001.pdf]

Angewandte Chemie
Eine Zeitschrift der Gesellschaft Deutscher Chemiker

Supporting Information

Methyl Substitution Destabilizes Alkyl Radicals

*E. Blokker, W.-J. van Zeist, X. Sun, J. Poater, J. M. van der Schuur, T. A. Hamlin, F. M. Bickelhaupt**

Contents

Computational Methods

Table S1. Homolytic bond dissociation enthalpies ΔH_{BDE} (in kcal mol^{-1}) for $\mathrm{Me_mH_{3\text{-}m}C}$–X with X = H, $\mathrm{CH_3}$, and OH.

Figure S1. Effect (in kcal mol^{-1}) of substituting hydrogens for m = 0, 3 methyl groups on ΔH_{Par}(X,m) and ΔH_{Rad}(m) in Scheme 2 for X = H and OH. Computed at Method/TZ2P//M06-2X/TZ2P (298.15 K and 1 atm) with Method a) BLYP, b) BP86, c) PBE, d) B3LYP, e) PBE0, f) ωB97, g) B2PLYP, h) B2TPLYP and i) rev-DSD-BLYP.

Table S2. $\mathrm{Me_mH_{3\text{-}m}C}$–X (m = 0, 3 for X = H, OH) bond dissociation enthalpies (ΔH_{BDE}) and ΔH_{Par}(X,m) and ΔH_{Rad}(m) of the thermochemical cycle in Scheme 2 (in kcal mol^{-1}).

Figure S2. a-b) Activation strain analysis and c-d) energy decomposition analysis as a function of the C–X distance (in kcal mol^{-1}) of the homolytic $\mathrm{Me_mH_{3\text{-}m}C}$–X bond dissociation reaction for m = 0, 3 and a,c) X = H and b,d) X = OH. Computed at M06-2X/TZ2P at the equilibrium geometries.

Table S3. Substituent–carbon bond distances (in Å) of $\mathrm{R_3CX}$ or $\mathrm{R_3C^{\bullet}}$, where R = H, Me and with X = H, OH.

Table S4. $\mathrm{Me_mH_{2\text{-}m}C}$–Y homolytic bond dissociation enthalpies ΔH_{BDE} (BDEs) of the alkyl radicals.

Figure S3. Effect (in kcal mol^{-1}) of substituting hydrogens for m = 0 – 3 methyl groups on ΔE_{Par}(X,m), the reaction m $\mathrm{Me^{\bullet}}$ + (3–m) $\mathrm{H^{\bullet}}$ + $\mathrm{CX^{\bullet\bullet\bullet}}$ → $\mathrm{Me_mH_{3\text{-}m}CX}$, and ΔE_{Rad}(m), the reaction m $\mathrm{Me^{\bullet}}$ + (3–m) $\mathrm{H^{\bullet}}$ + $\mathrm{C^{\bullet\bullet\bullet\bullet}}$ → $\mathrm{Me_mH_{3\text{-}m}C^{\bullet}}$ with X = H, OH. Computed at M06-2X/TZ2P at the equilibrium geometries.

Table S5. Activation strain analyses (in kcal mol^{-1}, Å) on $\Delta E_{\mathrm{R\text{–}R\text{–}R}}$, the reaction 3 $\mathrm{R^{\bullet}}$ → $\mathrm{R_3^{\bullet\bullet\bullet}}$, where $\mathrm{R_3^{\bullet\bullet\bullet}}$ is in the geometry of $\mathrm{R_3CX}$ or $\mathrm{R_3C^{\bullet}}$ (left side of the Table figure), and on ΔE_{Par}(X), the reaction 3 $\mathrm{R^{\bullet}}$ + $\mathrm{CX^{\bullet\bullet\bullet}}$ → $\mathrm{R_3CX}$, and on ΔE_{Rad}, the reaction 3 $\mathrm{R^{\bullet}}$ + $\mathrm{C^{\bullet\bullet\bullet\bullet}}$ → $\mathrm{R_3C^{\bullet}}$ (right side of the Table figure), where R = H, Me and with X = H, OH.

Table S6. Activation strain and energy decomposition analysis (in kcal mol^{-1}, Å) on ΔE_{Par}(X,m), the reaction m $\mathrm{Me^{\bullet}}$ + (3–m) $\mathrm{H^{\bullet}}$ + $\mathrm{CX^{\bullet\bullet\bullet}}$ → $\mathrm{Me_mH_{3\text{-}m}CX}$, and on ΔE_{Rad}(m), the reaction m $\mathrm{Me^{\bullet}}$ + (3–m) $\mathrm{H^{\bullet}}$ + $\mathrm{C^{\bullet\bullet\bullet\bullet}}$ → $\mathrm{Me_mH_{3\text{-}m}C^{\bullet}}$, where m = 0-3 and with X = H, OH.

Table S7. VDD charges (in milli-electrons) in $\mathrm{Me_mH_{3\text{-}m}CX}$ and $\mathrm{Me_mH_{3\text{-}m}C^{\bullet}}$ in terms of $\mathrm{Me_mH_{3\text{-}m}^{\bullet\bullet\bullet}}$ interacting with $\mathrm{CX^{\bullet\bullet\bullet}}$ or $\mathrm{C^{\bullet\bullet\bullet\bullet}}$, where m = 0, 3 and with X = H, OH.

Figure S4. Effect (in kcal mol^{-1}) of substituting hydrogens for m = 0 – 3 methyl groups on ΔE_{Par}(X,m), the reaction m $\mathrm{Me^{\bullet}}$ + (3–m) $\mathrm{H^{\bullet}}$ + $\mathrm{CX^{\bullet\bullet\bullet}}$ → $\mathrm{Me_mH_{3\text{-}m}CX}$, and ΔE_{Rad}(m), the reaction m $\mathrm{Me^{\bullet}}$ + (3–m) $\mathrm{H^{\bullet}}$ + $\mathrm{C^{\bullet\bullet\bullet\bullet}}$ → $\mathrm{Me_mH_{3\text{-}m}C^{\bullet}}$, and their corresponding activation strain analysis, with X = H, OH. Computed at M06-2X/TZ2P and, for each m, at equal substituent–carbon distances based on the geometry of $\mathrm{Me_mH_{3\text{-}m}C^{\bullet}}$.

Figure S5. Effect (in kcal mol^{-1}) of substituting hydrogens for m = 0 – 3 methyl groups on the energy decomposition analysis of ΔE_{Par}(X,m), the reaction m $\mathrm{Me^{\bullet}}$ + (3–m) $\mathrm{H^{\bullet}}$ + $\mathrm{CX^{\bullet\bullet\bullet}}$ → $\mathrm{Me_mH_{3\text{-}m}CX}$, and of ΔE_{Rad}(m), the reaction m $\mathrm{Me^{\bullet}}$ + (3–m) $\mathrm{H^{\bullet}}$ + $\mathrm{C^{\bullet\bullet\bullet\bullet}}$ → $\mathrm{Me_mH_{3\text{-}m}C^{\bullet}}$, with X = H, OH. Computed at M06-2X/TZ2P and, for each m, at equal substituent–carbon distances based on the geometry of $\mathrm{Me_mH_{3\text{-}m}C^{\bullet}}$.

Table S8. Orbital interaction ΔE_{oi} (in kcal mol^{-1}) of $\mathrm{Me_mH_{3\text{-}m}CH}$ and $\mathrm{Me_mH_{3\text{-}m}C^{\bullet}}$ in terms of $\mathrm{Me_mH_{3\text{-}m}^{\bullet\bullet\bullet}}$ interacting with $\mathrm{CX^{\bullet\bullet\bullet}}$ or $\mathrm{C^{\bullet\bullet\bullet\bullet}}$ where m = 0, 3. Furthermore, the difference in orbital interaction $\Delta\Delta E_{\mathrm{oi}}$ at m = 3 with respect to m = 0 is given.

Figure S6. MO diagram in A_1' and A_2'' symmetry for a) $\mathrm{H_3C^{\bullet}}$, and in A_1 symmetry for b) $\mathrm{Me_3C^{\bullet}}$, c) $\mathrm{H_3CH}$ and d) $\mathrm{Me_3CH}$ in terms of $\mathrm{R_3^{\bullet\bullet\bullet}}$ interacting with $\mathrm{C^{\bullet\bullet\bullet\bullet}}$ or $\mathrm{CH^{\bullet\bullet\bullet}}$. Interactions: 2c–2e^{-} in black, 2c–3e^{-} in red, 2c–3e^{-} hyperconjugation between $\mathrm{Me_3^{\bullet\bullet\bullet}}$ $\sigma_{\mathrm{C\text{–}H}}$ and $\mathrm{C^{\bullet\bullet\bullet\bullet}}$ p SOMO or $\mathrm{CH^{\bullet\bullet\bullet}}$ p-type SOMO in blue. Computed at M06-2X/TZ2P at the equilibrium geometries.

Figure S7. MO diagram in E symmetry for a) $H_3C^{•}$ and b) $Me_3C^{•}$. Interactions: 2c–2e^{-} in black, 2c–3e^{-} in red, 2c–3e^{-} hyperconjugation between $Me_3^{•••}$ $\sigma_{C–H}$ and the $C^{••••}$ or $CH^{•••}$ p SOMO in blue. Computed at M06-2X/TZ2P at the equilibrium geometries.

Figure S8. MO diagram in E symmetry for a) H_3CH and b) Me_3CH. Interactions: 2c–2e^{-} in black, 2c–3e^{-} in red, 2c–3e^{-} hyperconjugation between $Me_3^{•••}$ $\sigma_{C–H}$ and $C^{••••}$ or $CH^{•••}$ p SOMO in blue. Computed at M06-2X/TZ2P at the equilibrium geometries.

Figure S9. Schematic MO diagram for $Me_mH_{3-m}C^{•}$ of the 2c–3e^{-} hyperconjugation between $Me_mH_{3-m}^{•••}$ $\sigma_{C–H}$ and $C^{••••}$ p SOMO, where m = 0-3. Computed at M06-2X/TZ2P at the equilibrium geometries.

Table S9. Overlap S and energy gap $\Delta\varepsilon$ (in eV) of the 2c–2e^{-} interactions in $Me_mH_{3-m}CX$ and in $Me_mH_{3-m}C^{•}$ in terms of $Me_mH_{3-m}^{•••}$ interacting with $CX^{•••}$ or $C^{••••}$, where m = 0-3 and X = H, OH.

Table S10. Overlap S and energy gap $\Delta\varepsilon$ (in eV) of the 2c–3e^{-} hyperconjugation in $Me_mH_{3-m}CX$ and in $Me_mH_{3-m}C^{•}$ between $Me_mH_{3-m}^{•••}$ $\sigma_{C–H}$ and $CX^{•••}$ or $C^{••••}$ p SOMO, where X = H, OH and m = 0, 3.

Table S11. Overlap S and energy gap $\Delta\varepsilon$ (in eV) of 2c–3e^{-} interactions in $Me_mH_{3-m}CX$ or $Me_mH_{3-m}C^{•}$ in terms of $Me_mH_{3-m}^{•••}$ interacting with $CX^{•••}$ or $C^{••••}$, where X = H, OH and m = 0, 3.

Table S12. Overlap S and energy gap $\Delta\varepsilon$ (in eV) of 2c–4e^{-} interactions in Me_3CX in terms of $Me_3^{•••}$ interacting with $CX^{•••}$, where X = H, OH.

Figure S10. NBO orbitals involved in the hyperconjugation interaction between $\sigma_{C–H}$ (bottom) and $\sigma^*_{C–H}$, $\sigma^*_{C–O}$ or $2p_z$ SOMO (top) in Me_3CH, Me_3COH and $Me_3C\cdot$, respectively. Computed at M06-2X/cc-pVTZ//M06-2X/TZ2P level at the equilibrium geometries by means of NBO 6.0.

Figure S11. Schematic NBO orbital interaction diagram for Me_3CH, Me_3COH and $Me3C^{•}$ of the 2c–3e^{-} hyperconjugation between $\sigma_{C–H}$ and $\sigma^*_{C–H}$, $\sigma^*_{C–O}$, or $2p_z$ SOMO. Second-order perturbative estimates of the interactions in the NBO basis $E(2)$ (in kcal/mol), occupancies of the donor and acceptor NBOs O(don) and O(acc) (in a.u.), and NBO energy gaps $\Delta\varepsilon$ (in eV) are enclosed. Computed at M06-2X/cc-pVTZ//M06-2X/TZ2P level at the equilibrium geometries by means of NBO 6.0.

Table S13. Cartesian coordinates (in Å), ADF total energies [in brackets] (in kcal mol^{-1}), and number of imaginary frequencies NIMAG of the equilibrium geometries of all closed-shell species in this study, computed at M06-2X/TZ2P.

Table S14. Cartesian coordinates (in Å), ADF total energies [in brackets] (in kcal mol^{-1}), and number of imaginary frequencies NIMAG of the equilibrium geometries of all radical species in this study, computed at M06-2X/TZ2P.

Computational Methods
Computational details

All calculations were performed with the Amsterdam Density Functional (ADF) program unless otherwise stated.[1,2] Molecular orbitals (MOs) were expanded using a large uncontracted set of Slater-type orbitals (STO): TZ2P.[3] The TZ2P basis set is of triple-ζ quality, augmented by two sets of polarization functions. All electrons were treated variationally. The meta-hybrid generalized gradient approximation (GGA) functional M06-2X was used for calculating the geometries and energies.[4] M06-2X was chosen based on earlier works in which the performance of density functional methods was investigated on trends in R–X bond dissociation energies with R = Me, Et, *i*-Pr, *t*-Bu and various X (for instance H, CH_3, Cl, and OH).[5,6] In addition, our computed values with M06-2X nicely recover experimental R–X BDE values (see Table S1 and relevant references therein). Our conclusion from the analyses at M06-2X/TZ2P, that both the radical and parent molecule are destabilized upon methyl substitution, is nicely reproduced when carried out with nine other functionals (in combination with the same TZ2P basis set), namely: a) BLYP, b) BP86, c) PBE, d) B3LYP, e) PBE0, f) ωB97, g) B2PLYP, h) B2TPLYP and i) rev-DSD-BLYP (see Table S2 and Figure S1).[7] No geometry restrictions were used unless otherwise stated. The radical fragments were treated spin-unrestricted and the PyFrag2019 program was used for analyzing the bond dissociation as a function of the $Me_mH_{3-m}C$–X distance.[8] For ease, and only in the case of displaying overlaps, fragments without spin polarization are used. NBO analyses were performed with the Gaussian 09 rev. D01 program at M06-2X/cc-pVTZ level of theory on the geometries optimized at M06-2X/TZ2P in ADF to highlight the hyperconjugation interaction in the parent molecule and the radical (see Figures S10-S11).[9,10]

Thermochemistry

Enthalpies at 298.15 K and 1 atmosphere (ΔH_{298}) were calculated from electronic bond energies (ΔE) and vibrational frequencies using standard thermochemistry relations for an ideal gas, according to Equation (2):[11]

$$\Delta H_{298} = \Delta E + \Delta E_{\text{trans},298} + \Delta E_{\text{rot},298} + \Delta E_{\text{vib},0} + \Delta(\Delta E_{\text{vib},0})_{298} + \Delta(pV) \qquad (2)$$

Here, $\Delta E_{\text{trans},298}$, $\Delta E_{\text{rot},298}$ and $\Delta E_{\text{vib},0}$ are the differences between the reactant and products in translational, rotational and zero-point vibrational energy, respectively. $\Delta(\Delta E_{\text{vib},0})_{298}$ is the change in the vibrational energy difference as one goes from 0 to 298.15 K. The vibrational energy corrections are identical to our frequency calculations. The molar work term $\Delta(pV)$ is $(\Delta n)RT$; $\Delta n = +1$ for one reactant dissociating into the two products. Thermal corrections for the electronic energy are neglected.

Activation strain and energy decomposition analysis

For the activation strain analysis (ASA), the bond energy ΔE [which also features in Eq. (2)] between two fragments is made up of two major components:[12]

$$\Delta E = \Delta E_{\text{strain}} + \Delta E_{\text{int}} \qquad (3)$$

Here, the strain energy ΔE_{strain} is the amount of energy required to deform the fragments from their equilibrium structure to the geometry that they acquire in the overall complex. The interaction energy ΔE_{int} corresponds to the actual energy change when the geometrically deformed fragments are combined to form the overall complex.

We further analyze the interaction ΔE_{int} in the framework of the canonical Kohn-Sham molecular orbital (MO) model, by dissecting it through our canonical energy decomposition analyses (canonical EDA) into the electrostatic attraction, the Pauli repulsion and the (attractive) orbital interactions:[1, 12]

$$\Delta E_{\text{int}} = \Delta V_{\text{elstat}} + \Delta E_{\text{Pauli}} + \Delta E_{\text{oi}} \tag{4}$$

The term ΔV_{elstat} corresponds to the classical electrostatic interaction between the unperturbed charge distributions of the fragments in the geometry they possess in the complex. This term is usually attractive. The Pauli-repulsion ΔE_{Pauli} between these fragments comprises the destabilizing interactions, associated with the Pauli-principle for fermions, between occupied orbitals and is responsible for the steric repulsion. The orbital interaction ΔE_{oi} between these fragments in any MO model, and therefore also in Kohn-Sham theory, accounts for electron-pair bonding (the SOMO–SOMO interaction), charge transfer (empty/occupied orbital mixing between different fragments) and polarization (empty/occupied orbital mixing on one fragment due to the presence of another fragment). The orbital interaction energy ΔE_{oi} can be further decomposed into the contributions from each irreducible representation Γ of the interacting system. The use of M06-2X gives a term that cannot be decomposed, which is a correction term, such that the total orbital interaction is the correct one.

Voronoi Deformation Density (VDD) Charge

The electron density distribution is analyzed by using the Voronoi deformation density (VDD) method for atomic charges.[13] The VDD atomic charge $Q_{\text{A}}^{\text{VDD}}$ is computed as the (numerical) integral of the deformation density $\Delta\rho(\mathbf{r}) = \rho(\mathbf{r}) - \sum_{\text{B}} \rho_{\text{B}}(\mathbf{r})$ in the volume of the Voronoi cell of atom A [Eq. (7)].[14] The Voronoi cell of atom A is defined as the compartment of space bound by the bond midplanes on and perpendicular to all bond axes between nucleus A and its neighboring nuclei (cf. the Wigner-Seitz cells in crystals).[13]

$$Q_{\text{A}}^{\text{VDD}} = -\int_{\text{Voronoi cell of A}} \left[\rho(\mathbf{r}) - \textstyle\sum_{\text{B}} \rho_{\text{B}}(\mathbf{r})\right] d\mathbf{r} \tag{5}$$

In Eq. (5), $\rho(\mathbf{r})$ is the electron density of the molecule and $\sum_{\text{B}} \rho_{\text{B}}(\mathbf{r})$ the superposition of atomic densities ρ_{B} of a fictitious promolecule without chemical interactions that is associated with the situation in which all atoms are neutral. The interpretation of the VDD charge $Q_{\text{A}}^{\text{VDD}}$ is rather straightforward and transparent. Instead of measuring the amount of charge associated with a particular atom A, $Q_{\text{A}}^{\text{VDD}}$ directly monitors how much charge flows, due to chemical interactions, out of ($Q_{\text{A}}^{\text{VDD}} > 0$) or into ($Q_{\text{A}}^{\text{VDD}} < 0$) the Voronoi cell of atom A, that is, the region of space that is closer to nucleus A than to any other nucleus.

References

[1] ADF2019.305, SCM, Theoretical Chemistry, Vrije Universiteit Amsterdam (The Netherlands), http://www.scm.com.

[2] G. te Velde, F. M. Bickelhaupt, E. J. Baerends, C. Fonseca Guerra, S. J. A. van Gisbergen, J. G. Snijders, T. Ziegler, *J. Comput. Chem.* **2001**, *22*, 931.

[3] E. van Lenthe, E. J. Baerends, *J. Comput. Chem.* **2003**, *24*, 1142.

[4] Y. Zhao, D. G. Truhlar, *Theor. Chem. Acc.* **2008**, *120*, 215.

[5] E. I. Izgorodina, M. L. Coote, L. Radom, *J. Phys. Chem. A* **2005**, *109*, 7558–7566.

[6] I. Y. Zhang, J. Wu, Y. Luo, X. Xu, *J. Chem. Theory Comput.* **2010**, *6*, 1462.

[7] a) A. D. Becke, *Phys. Rev. A.* **1988**, *38*, 3098; b) C. T. Lee, W. T. Yang, R. G. Parr, *Phys. Rev. B.* **1988**, *37*, 785; c) J. P. Perdew, *Phys. Rev. B* **1986**, *33*, 8822. Erratum: J. P. Perdew, *Phys. Rev. B* **1986**, *34*, 7406; d) J. P. Perdew, K. Burke, M. Ernzerhof, *Phys. Rev. Lett.* **1996**, *77*, 3865; e) P. J. Stephens, F. J. Devlin, C. F. Chabalowski, M. J. Frisch, *J. Phys. Chem.* **1994**, *98*, 11623; f) S. Grimme, *J. Comput. Chem.* **2004**, *25*, 1463; g) M. Ernzerhof, G. Scuseria, *J. Chem. Phys.* **1999**, *110*, 5029; h) J.-D. Chai, M. Head-Gordon, *J. Chem. Phys.* **2008**, *128*, 084106; i) S. Grimme, *J. Chem. Phys.* **2006**, *124*, 034108; j) A. Tarnopolsky, A. Karton, R. Sertchook, D. Vuzman, J. M. L Martin, *J. Phys. Chem. A* **2008**, *112*, 3; k) G. Santra, N. Sylvetsky, J. M. L. Martin, *J. Chem. Phys.* **2019**, *123*, 5129.

[8] a) W. J. van Zeist, C. Fonseca Guerra, F. M. Bickelhaupt, *J. Comp. Chem.* **2008**, *29*, 312; b) X. Sun, T. M. Soini, J. Poater, T. A. Hamlin, F. M. Bickelhaupt, *J. Comput. Chem.* **2019**, *40*, 2227; c) PyFrag 2007–2021: X. Sun, T. Soini, L. P. Wolters, W.-J. van Zeist, C. Fonseca Guerra, T. A. Hamlin, F. M. Bickelhaupt, Vrije Universiteit Amsterdam, The Netherlands.

[9] M. J. Frisch, G. W. Trucks, H. B. Schlegel, G. E. Scuseria, M. A. Robb, J. R.Cheeseman, G. Scalmani, V. Barone, B. Mennucci, G. A. Petersson, H. Nakatsuji, M. Caricato, X. Li, H. P. Hratchian, A. F. Izmaylov, J. Bloino, G. Zheng, J. L. Sonnenberg, M. Hada, M. Ehara, K. Toyota, R. Fukuda, J. Hasegawa, M. Ishida, T. Nakajima, Y. Honda, O. Kitao, H. Nakai, T. Vreven, J. A. Montgomery Jr., J. E. Peralta, F. Ogliaro, M. Bearpark, J. J. Heyd, E. Brothers, K. N. Kudin, V. N. Staroverov, R. Kobayashi, J. Normand, K. Raghavachari, A. Rendell, J. C. Burant, S. S. Iyengar, J. Tomasi, M. Cossi, N. Rega, J. M. Millam, M. Klene, J. E. Knox, J. B. Cross, V. Bakken, C. Adamo, J. Jaramillo, R. Gomperts, R. E. Stratmann, O. Yazyev, A. J. Austin, R. Cammi, C. Pomelli, J. W. Ochterski, R. L. Martin, K. Morokuma, V. G. Zakrzewski, G. A. Voth, P. Salvador, J. J. Dannenberg, S. Dapprich, A. D. Daniels, Ö. Farkas, J. B. Foresman, J. V. Ortiz, J. Cioslowski, D. J. Fox, Gaussian 09, Revision D.01; Gaussian, Inc., Wallingford CT, 2013.

[10] a) E. D. Glendening, J. K. Badenhoop, A. E. Reed, J. E. Carpenter, J. A. Bohmann, C. M. Morales, C. R. Landis, F. Weinhold, *NBO 6.0*. Theoretical Chemistry Institute, University of Wisconsin, Madison, **2013**; b) E. D. Glendening, C. R. Landis, F. Weinhold, *J. Comput. Chem.* **2013**, *34*, 1429.

[11] a) P. W. Atkins, J. de Paula, *Physical Chemistry, 9th ed.*, W. H. Freeman, New York, **2010**; b) F. Jensen, *Introduction to Computational Chemistry*, Wiley, **2007**.

[12] a) T. A. Hamlin, P. Vermeeren, C. Fonseca Guerra, F. M. Bickelhaupt, in *Complementary Bonding Analysis* (Ed: S. Grabowsky), De Gruyter, Berlin, Boston, **2021**, pp 199–212; b) F. M. Bickelhaupt, E. J. Baerends, in *Reviews in Computational Chemistry*, (Eds.: K. B. Lipkowitz, D. B. Boyd), Wiley–VCH, Hoboken, **2000**, pp 1–86; c) F. M. Bickelhaupt, N. M. M. Nibbering, E. M. van Wezenbeek, E. J. Baerends, *J. Phys. Chem.* **1992**, *96*, 4864; d) T. Ziegler, A. Rauk, *Theoret. Chim. Acta.* **1977**, *46*, 1; e) T. Ziegler, A. Rauk, *Inorg. Chem.* **1979**, *18*, 1755.

[13] a) C. Fonseca Guerra, F. M. Bickelhaupt, J. G. Snijders, E. J. Baerends, *Chem. Eur. J.* **1999**, *5*, 3581; b) C. Fonseca Guerra, J. W. Handgraaf, E. J. Baerends, F. M. Bickelhaupt, *J. Comput. Chem.* **2004**, *25*, 189; c) O. A. Stasyuk, H. Szatylowicz, T. M. Krygowski, C. Fonseca Guerra, *Phys. Chem. Chem. Phys.* **2016**, *18*, 11624.

[14] G. te Velde, E. J. Baerends, *J. Comput. Phys.* **1992**, *99*, 84.

Table S1. Homolytic bond dissociation enthalpies ΔH_{BDE} (in kcal mol^{-1}) for $Me_mH_{3-m}C$–X with X = H, CH_3, and OH.

$Me_mH_{3-m}C$–X	m	M06-2X/TZ2P[a]	G4[b]	Experimental[c,d]
H_3C–H	0	103.7	104.5	104.81
MeH_2C–H	1	99.8	100.7	100.6 ± 0.6
Me_2HC–H	2	96.9	97.9	98.1 ± 0.6
Me_3C–H	3	95.2	96.0	95.2 ± 0.9
H_3C–CH_3	0	90.0	89.0	89.7 ± 0.1
MeH_2C–CH_3	1	88.5	88.0	88.2 ± 0.6
Me_2HC–CH_3	2	87.3	87.3	87.9 ± 0.7
Me_3C–CH_3	3	86.3	86.5	86.0 ± 0.9
H_3C–OH	0	92.8	91.1	93.1 ± 2.4, 91.9 ± 1.0[e]
MeH_2C–OH	1	94.2	93.1	93.7 ± 1.0
Me_2HC–OH	2	95.3	94.5	95.5 ± 0.5
Me_3C–OH	3	95.8	95.3	95.0 ± 0.9

[a] This work, computed at 298.15 K and 1 atm at the equilibrium geometries. [b] Reference 6. [c] Reference 15. [d] Heat of formation of isopropyl radical $Me_2HC^{\bullet}$ from Reference 16. [e] Reference 17.

References

[15] National Institute for Standards and Technology (NIST). Chemistry Webbook. https://webbook.nist.gov/chemistry (accessed January 4, 2021).

[16] W. Tsang, in *Energetics of Organic Free Radicals*, (Eds.: J. A. M. Simões, A. Greenberg, J. F. Liebman), Blackie Academic and Professional, London, **1996**, pp 22–58.

[17] Active Thermochemical Tables. https://atct.anl.gov/Thermochemical%20Data/version%201.122p (accessed January 4, 2021).

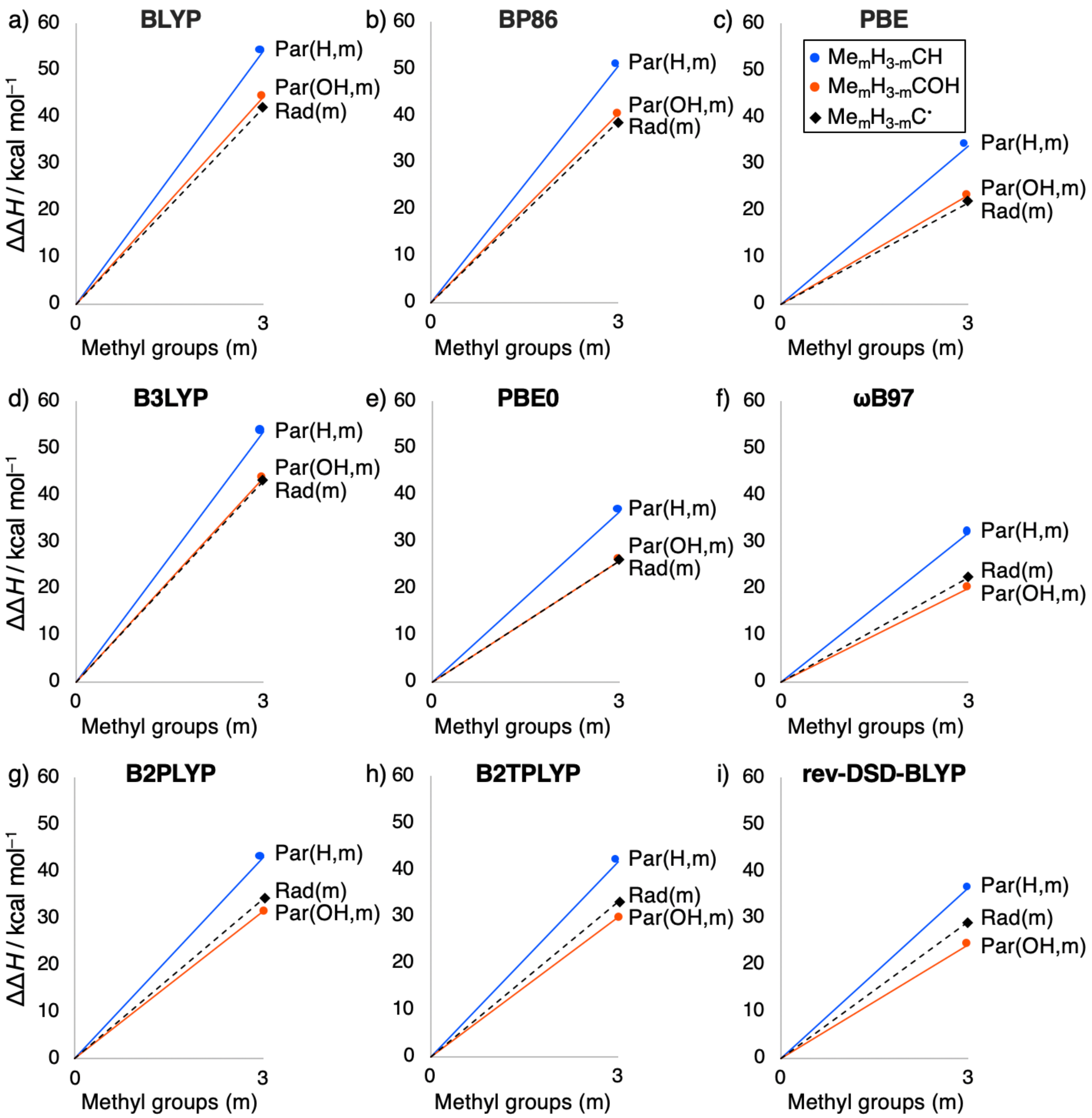

Figure S1. Effect (in kcal mol^{-1}) of substituting hydrogens for m = 0, 3 methyl groups on ΔH_{Par}(X,m) and ΔH_{Rad}(m) in Scheme 2 for X = H and OH. Computed at Method/TZ2P//M06-2X/TZ2P (298.15 K and 1 atm) with Method being: a) BLYP, b) BP86, c) PBE, d) B3LYP, e) PBE0, f) ωB97, g) B2PLYP, h) B2TPLYP, and i) rev-DSD-BLYP.

Table S2. $Me_mH_{3-m}C$–X (m = 0, 3 for X = H, OH) bond dissociation enthalpies (ΔH_{BDE}) and ΔH_{Par}(X,m) and ΔH_{Rad}(m) of the thermochemical cycle in Scheme 2 (in kcal mol^{-1}).[a]

Method		BLYP			BP86		
**$Me_mH_{3-m}C$–X**	**m**	**ΔH_{BDE}**	**ΔH_{Par}(X,m)**	**ΔH_{Rad}(m)**	**ΔH_{BDE}**	**ΔH_{Par}(X,m)**	**ΔH_{Rad}(m)**
H_3C–H	0	101.8	–334.5	–402.9	104.0	–337.1	–401.0
Me_3C–H	3	89.8	–280.5	–360.9	92.0	–286.5	–362.4
H_3C–OH	0	87.2	–338.0	–402.9	90.6	–340.7	–401.0
Me_3C–OH	3	85.1	–293.9	–360.9	88.8	–300.4	–362.4

Method		PBE			B3LYP		
**$Me_mH_{3-m}C$–X**	**m**	**ΔH_{BDE}**	**ΔH_{Par}(X,m)**	**ΔH_{Rad}(m)**	**ΔH_{BDE}**	**ΔH_{Par}(X,m)**	**ΔH_{Rad}(m)**
H_3C–H	0	102.0	–329.7	–389.2	102.7	–336.2	–402.4
Me_3C–H	3	89.9	–296.1	–367.6	92.0	–282.5	–359.5
H_3C–OH	0	93.3	–333.1	–389.2	86.3	–341.4	–402.4
Me_3C–OH	3	91.7	–309.9	–367.6	85.6	–297.8	–359.5

Method		PBE0			ωB97		
**$Me_mH_{3-m}C$–X**	**m**	**ΔH_{BDE}**	**ΔH_{Par}(X,m)**	**ΔH_{Rad}(m)**	**ΔH_{BDE}**	**ΔH_{Par}(X,m)**	**ΔH_{Rad}(m)**
H_3C–H	0	101.4	–327.7	–386.1	103.7	–336.9	–394.2
Me_3C–H	3	90.8	–291.2	–360.2	94.3	–305.1	–371.8
H_3C–OH	0	89.1	–332.8	–386.1	91.5	–344.3	–394.2
Me_3C–OH	3	89.1	–306.9	–360.2	93.9	–324.2	–371.8

Method		B2PLYP			B2TPLYP		
**$Me_mH_{3-m}C$–X**	**m**	**ΔH_{BDE}**	**ΔH_{Par}(X,m)**	**ΔH_{Rad}(m)**	**ΔH_{BDE}**	**ΔH_{Par}(X,m)**	**ΔH_{Rad}(m)**
H_3C–H	0	102.8	–333.3	–395.1	102.7	–332.5	–393.2
Me_3C–H	3	93.9	–290.3	–361.0	94.2	–290.9	–360.1
H_3C–OH	0	89.6	–339.9	–395.1	89.6	–339.5	–393.2
Me_3C–OH	3	92.1	–308.4	–361.0	92.7	–309.5	–360.1

Method		rev-DSD-BLYP		
**$Me_mH_{3-m}C$–X**	**m**	**ΔH_{BDE}**	**ΔH_{Par}(X,m)**	**ΔH_{Rad}(m)**
H_3C–H	0	103.6	–334.7	–394.5
Me_3C–H	3	96.3	–298.3	–365.3
H_3C–OH	0	91.3	–342.8	–394.5
Me_3C–OH	3	96.2	–318.5	–365.3

[a] Computed at Method/TZ2P//M06-2X/TZ2P at 298.15 K and 1 atm.

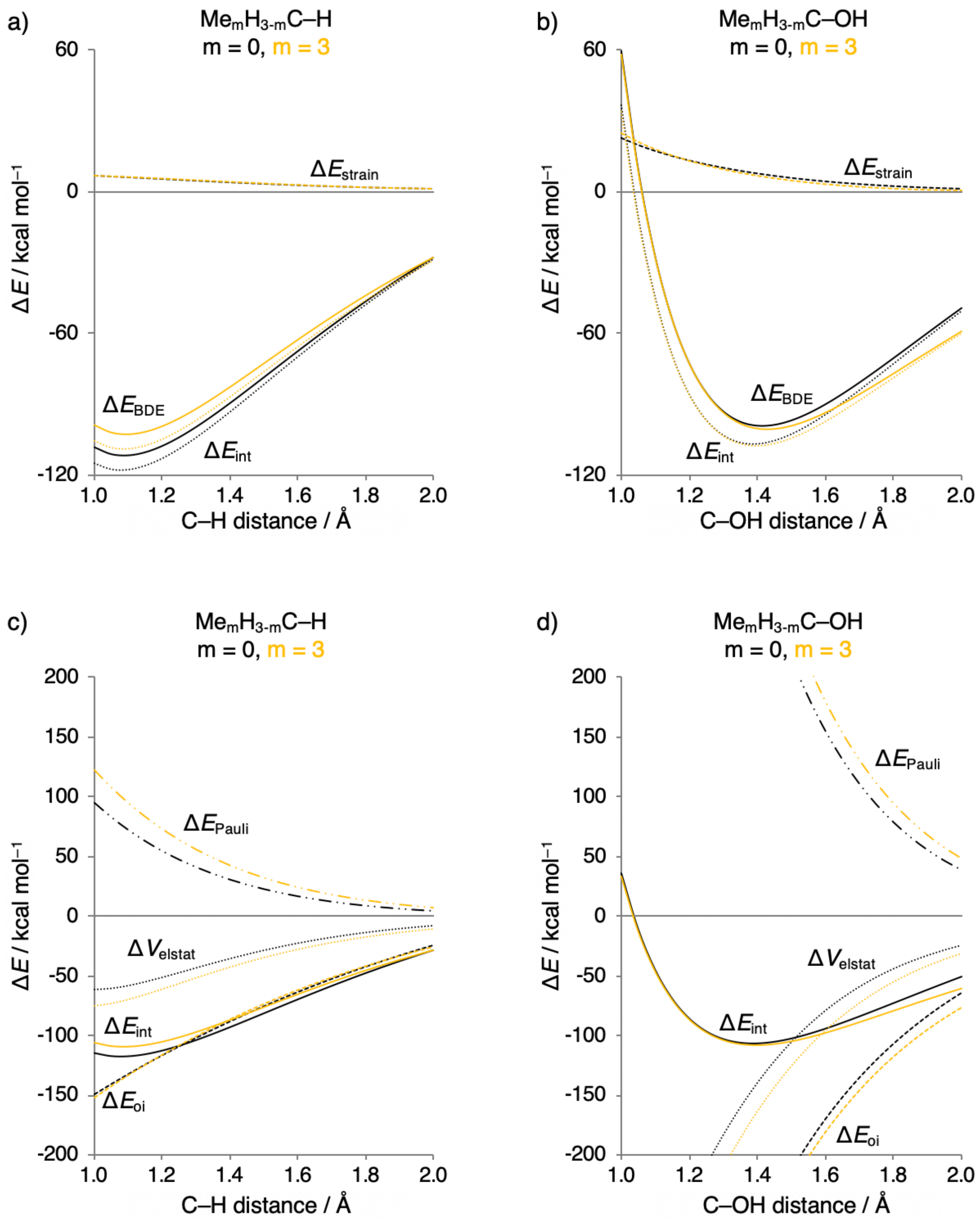

Figure S2. a-b) Activation strain analysis and c-d) energy decomposition analysis as a function of the C–X distance (in kcal mol^{-1}) of the homolytic $Me_mH_{3-m}C$–X bond dissociation reaction for m = 0, 3 and a,c) X = H and b,d) X = OH. Computed at M06-2X/TZ2P at the equilibrium geometries.

Table S3. Substituent–carbon bond distances (in Å) of R_3CX or $R_3C^•$, where R = H, Me and with X = H, OH.[a]

		$d_{(R–C)}$	$d_{(R–C)}$	$d_{(R–C)}$
**Me_mH_{3-m}–CH**				
H_3–CH	0	1.087	1.087	1.087
H_2Me–CH	1	1.089	1.089	1.525
HMe_2–CH	2	1.091	1.524	1.524
Me_3–CH	3	1.526	1.526	1.526
**Me_mH_{3-m}–COH**				
H_3–COH	0	1.087	1.092	1.092
H_2Me–COH	1	1.094	1.094	1.512
HMe_2–COH	2	1.096	1.516	1.520
Me_3–COH	3	1.521	1.526	1.526
**Me_mH_{3-m}–$C^•$**				
H_3–$C^•$	0	1.076	1.076	1.076
H_2Me–$C^•$	1	1.079	1.079	1.485
HMe_2–$C^•$	2	1.080	1.487	1.487
Me_3–$C^•$	3	1.491	1.491	1.491

[a] Computed at M06-2X/TZ2P at the equilibrium geometries.

Table S4. $Me_mH_{2-m}C^•$–X homolytic bond dissociation enthalpies ΔH_{BDE} (BDEs) of the alkyl radicals.

$Me_mH_{2-m}C^•$–X	m	X = H	X = CH_3
$H_2C^•$–X	0	107.5	97.7
$MeHC^•$–X	1	104.5	96.1
$Me_2C^•$–X	2	102.6	94.7

[a] This work, computed at M06-2X/TZ2P (298.15 K and 1 atm) at the equilibrium geometries.

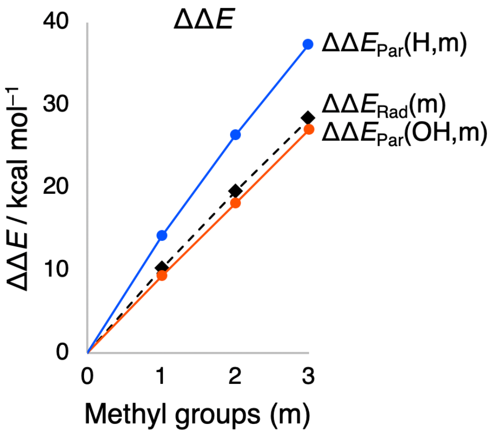

Figure S3. Effect (in kcal mol^{-1}) of substituting hydrogens for m = 0 – 3 methyl groups on ΔE_{Par}(X,m), the reaction m $Me^•$ + (3–m) $H^•$ + $CX^{•••}$ → $Me_mH_{3-m}CX$, and ΔE_{Rad}(m), the reaction m $Me^•$ + (3–m) $H^•$ + $C^{••••}$ → $Me_mH_{3-m}C^•$ with X = H, OH. Computed at M06-2X/TZ2P at the equilibrium geometries.

Table S5. Activation strain analyses (in kcal mol^{-1}, Å) on $\Delta E_{\mathrm{R-R-R}}$, the reaction 3 $R^{\bullet} \rightarrow R_3^{\bullet\bullet\bullet}$, where $R_3^{\bullet\bullet\bullet}$ is in the geometry of R_3CX or $R_3C^{\bullet}$ (left side of the Table figure), and on $\Delta E_{\mathrm{Par}}(X)$, the reaction 3 $R^{\bullet} + CX^{\bullet\bullet\bullet} \rightarrow R_3CX$, and on ΔE_{Rad}, the reaction 3 $R^{\bullet} + C^{\bullet\bullet\bullet\bullet} \rightarrow R_3C^{\bullet}$ (right side of the Table figure), where R = H, Me and with X = H, OH.[a,b]

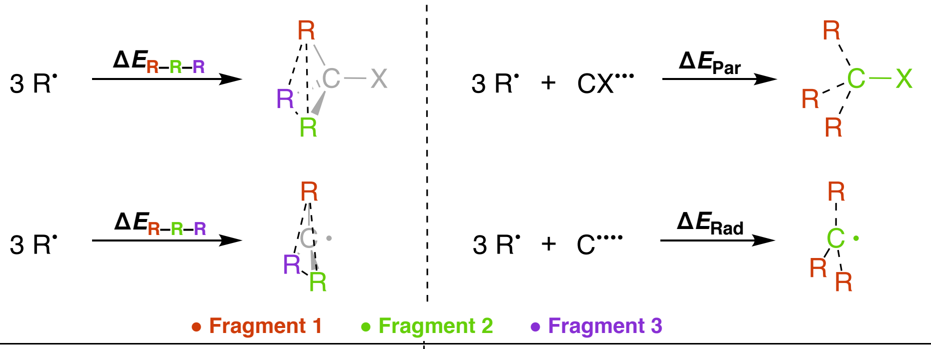

Energies (in kcal mol^{-1})	m	ΔE_{int}, R–R–R	$\Delta E_{\mathrm{strain}}$, R–R–R	ΔE, R–R–R	$\Delta E_{\mathrm{strain}}$, $R_3^{\bullet\bullet\bullet}$	$\Delta E_{\mathrm{strain}}$, $CX^{\bullet\bullet\bullet}$ or $C^{\bullet\bullet\bullet\bullet}$	$\Delta E_{\mathrm{strain}}$	ΔE_{int}	$\Delta E_{\mathrm{Par}}(X)$ / ΔE_{Rad}
**R_3–CH**									$\Delta E_{\mathrm{Par}}(X)$
H_3–CH	0	31.5	0.0	31.5	31.5	0.0	31.5	–383.0	–351.6
Me_3–CH	3	28.3	23.7	52.1	52.1	0.0	52.1	–366.3	–314.3
**R_3–COH**									$\Delta E_{\mathrm{Par}}(X)$
H_3–COH	0	32.1	0.0	32.1	32.1	1.5	33.6	–390.8	–357.2
Me_3–COH	3	28.8	21.9	50.7	50.7	2.1	52.8	–383.0	–330.2
**R_3–$C^{\bullet}$**									ΔE_{Rad}
H_3–$C^{\bullet}$	0	24.9	0.0	24.9	24.9	0.0	24.9	–439.6	–414.7
Me_3–$C^{\bullet}$	3	21.7	25.8	47.5	47.5	0.0	47.5	–432.7	–385.2

Distances for analysis (in Å)	m	$d_{\mathrm{(R-R)}}$	$d_{\mathrm{(R-R)}}$	$d_{\mathrm{(R-R)}}$	$d_{\mathrm{(R-C)}}$	$d_{\mathrm{(R-C)}}$	$d_{\mathrm{(R-C)}}$
**R_3–CH**							
H_3–CH	0	1.775	1.775	1.775	1.087	1.087	1.087
Me_3–CH	3	2.510	2.510	2.510	1.526	1.526	1.526
**R_3–COH**							
H_3–COH	0	1.763	1.763	1.770	1.087	1.087	1.087
Me_3–COH	3	2.511	2.511	2.510	1.526	1.526	1.526
**R_3–$C^{\bullet}$**							
H_3–$C^{\bullet}$	0	1.883	1.883	1.883	1.087	1.087	1.087
Me_3–$C^{\bullet}$	3	2.619	2.619	2.619	1.526	1.526	1.526

[a] Computed at M06-2X/TZ2P and, for each m, at equal substituent–carbon distances based on the geometry of $Me_mH_{3-m}C$–H. [b] A similar decomposition of the strain energy can be seen in: D. Rodrigues Silva, L. de Azevedo Santos, M. P. Freitas, C. Fonseca Guerra, T. A. Hamlin, *Chem. Asian. J.* **2020**, *15*, 4043.

Table S6. Activation strain and energy decomposition analysis (in kcal mol^{-1}, Å) on ΔE_{Par}(X,m), the reaction m Me$^{\bullet}$ + (3–m) H$^{\bullet}$ + CX$^{\bullet\bullet\bullet}$ → Me_mH_{3-m}CX, and on ΔE_{Rad}(m), the reaction m Me$^{\bullet}$ + (3–m) H$^{\bullet}$ + C$^{\bullet\bullet\bullet\bullet}$ → $Me_mH_{3-m}C^{\bullet}$, where m = 0-3 and with X = H, OH.[a]

	Energies (in kcal mol^{-1})								
	m	ΔV_{elstat}	ΔE_{Pauli}	ΔE_{oi}	ΔE_{int}	$\Delta E_{strain,\ R_3^{\bullet\bullet\bullet}}$	$\Delta E_{strain,\ CX^{\bullet\bullet\bullet}\ or\ C^{\bullet\bullet\bullet\bullet}}$	ΔE_{strain}	ΔE_{Par}(X,m) / ΔE_{Rad}(m)
**Me_mH_{3-m}–CH**									ΔE_{Par}(X,m)
H_3–CH	0	–127.2	131.2	–387.0	–383.0	31.5	0.0	31.5	–351.6
H_2Me–CH	1	–195.9	240.6	–420.0	–375.3	37.9	0.0	37.9	–337.4
HMe_2–CH	2	–263.3	350.5	–456.0	–368.8	43.6	0.0	43.6	–325.2
Me_3–CH	3	–329.3	457.1	–494.1	–366.3	52.1	0.0	52.1	–314.3
**Me_mH_{3-m}–COH**									ΔE_{Par}(X,m)
H_3–COH	0	–134.6	127.3	–383.5	–390.8	32.1	1.5	33.6	–357.2
H_2Me–COH	1	–204.5	233.9	–416.4	–387.0	37.4	1.7	39.1	–347.8
HMe_2–COH	2	–275.5	344.3	–452.3	–383.6	42.6	1.9	44.4	–339.1
Me_3–COH	3	–344.6	450.9	–489.3	–383.0	50.7	2.1	52.8	–330.2
**Me_mH_{3-m}–C$^{\bullet}$**									ΔE_{Rad}(m)
H_3–C$^{\bullet}$	0	–115.2	14.2	–338.6	–439.6	24.9	0.0	24.9	–414.7
H_2Me–C$^{\bullet}$	1	–179.6	114.6	–371.8	–436.9	32.9	0.0	32.9	–404.0
HMe_2–C$^{\bullet}$	2	–242.9	217.5	–408.3	–433.6	39.3	0.0	39.3	–394.3
Me_3–C$^{\bullet}$	3	–303.8	317.0	–445.8	–432.7	47.5	0.0	47.5	–385.2

	Distances (in Å)			
		$d_{(R–C)}$	$d_{(R–C)}$	$d_{(R–C)}$
**Me_mH_{3-m}–CH**				
H_3–CH	0	1.087	1.087	1.087
H_2Me–CH	1	1.089	1.089	1.525
HMe_2–CH	2	1.091	1.524	1.524
Me_3–CH	3	1.526	1.526	1.526
**Me_mH_{3-m}–COH**				
H_3–COH	0	1.087	1.087	1.087
H_2Me–COH	1	1.089	1.089	1.525
HMe_2–COH	2	1.091	1.524	1.524
Me_3–COH	3	1.526	1.526	1.526
**Me_mH_{3-m}–C$^{\bullet}$**				
H_3–C$^{\bullet}$	0	1.087	1.087	1.087
H_2Me–C$^{\bullet}$	1	1.089	1.089	1.525
HMe_2–C$^{\bullet}$	2	1.091	1.524	1.524
Me_3–C$^{\bullet}$	3	1.526	1.526	1.526

[a] Computed at M06-2X/TZ2P and, for each m, at equal substituent–carbon distances based on the geometry of Me_mH_{3-m}C–H.

Table S7. VDD charges (in milli-electrons) in $Me_mH_{3-m}CX$ and $Me_mH_{3-m}C^{\bullet}$ in terms of $Me_mH_{3-m}^{\bullet\bullet\bullet}$ interacting with $CX^{\bullet\bullet\bullet}$ or $C^{\bullet\bullet\bullet\bullet}$, where m = 0, 3 and with X = H, OH.[a]

	m	atoms H in $H_3^{\bullet\bullet\bullet}$ / atoms C in $Me_3^{\bullet\bullet\bullet}$	atom C in $CX^{\bullet\bullet\bullet}$ / $C^{\bullet\bullet\bullet\bullet}$
**Me_mH_{3-m}–CH**			
H_3–CH	0	0.0	–64.0
Me_3–CH	3	–37.0	–64.0
**Me_mH_{3-m}–COH**			
H_3–COH	0	0.0	–20.0
Me_3–COH	3	–38.0	–18.0
**Me_mH_{3-m}–$C^{\bullet}$**			
H_3–$C^{\bullet}$	0	0.0	0.0
Me_3–$C^{\bullet}$	3	–37.0	0.0

[a] Computed at M06-2X/TZ2P and, for each m, at equal substituent–carbon distances based on the geometry of $Me_mH_{3-m}C$–H.

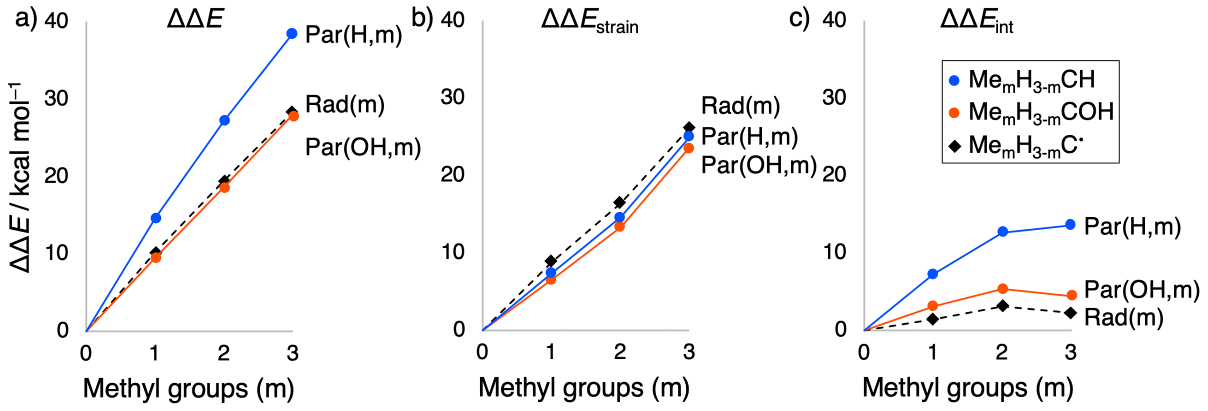

Figure S4. Effect (in kcal mol^{-1}) of substituting hydrogens for m = 0 – 3 methyl groups on ΔE_{Par}(X,m), the reaction m Me$^{\bullet}$ + (3–m) H$^{\bullet}$ + CX$^{\bullet\bullet\bullet}$ → Me$_{\mathrm{m}}$H$_{3\text{-m}}$CX, and ΔE_{Rad}(m), the reaction m Me$^{\bullet}$ + (3–m) H$^{\bullet}$ + C$^{\bullet\bullet\bullet\bullet}$ → Me$_{\mathrm{m}}$H$_{3\text{-m}}$C$^{\bullet}$, and their corresponding activation strain analysis, with X = H, OH. Computed at M06-2X/TZ2P and, for each m, at equal substituent–carbon distances based on the geometry of Me$_{\mathrm{m}}$H$_{3\text{-m}}$C$^{\bullet}$.

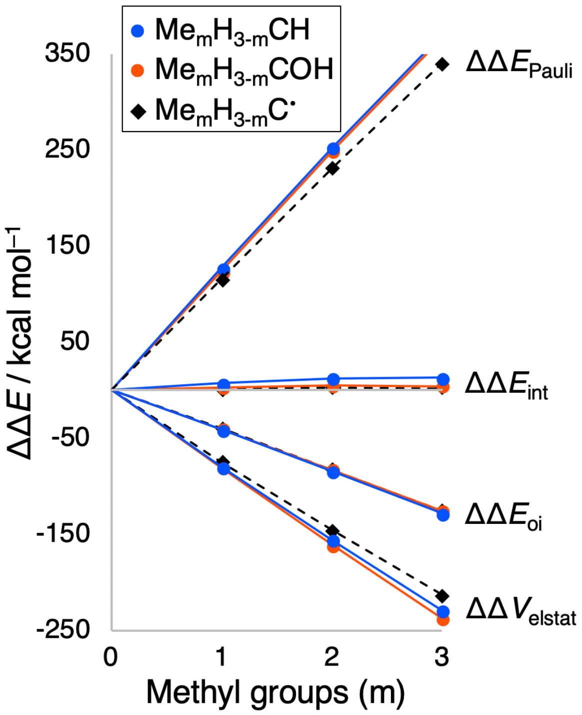

Figure S5. Effect (in kcal mol^{-1}) of substituting hydrogens for m = 0 – 3 methyl groups on the energy decomposition analysis of ΔE_{Par}(X,m), the reaction m Me$^{\bullet}$ + (3–m) H$^{\bullet}$ + CX$^{\bullet\bullet\bullet}$ → Me$_{\mathrm{m}}$H$_{3\text{-m}}$CX, and of ΔE_{Rad}(m), the reaction m Me$^{\bullet}$ + (3–m) H$^{\bullet}$ + C$^{\bullet\bullet\bullet\bullet}$ → Me$_{\mathrm{m}}$H$_{3\text{-m}}$C$^{\bullet}$, with X = H, OH. Computed at M06-2X/TZ2P and, for each m, at equal substituent–carbon distances based on the geometry of Me$_{\mathrm{m}}$H$_{3\text{-m}}$C$^{\bullet}$.

Table S8. Orbital interaction ΔE_{oi} (in kcal mol^{-1}) of $Me_mH_{3-m}CH$ and $Me_mH_{3-m}C^{\bullet}$ in terms of $Me_mH_{3-m}^{\bullet\bullet\bullet}$ interacting with $CX^{\bullet\bullet\bullet}$ or $C^{\bullet\bullet\bullet\bullet}$ where m = 0, 3. Furthermore, the difference in orbital interaction $\Delta\Delta E_{oi}$ at m = 3 with respect to m = 0 is given.[a]

	m	ΔE_{oi}	ΔE_{oi} a_1	ΔE_{oi} a_2	ΔE_{oi} e	Correction	$\Delta\Delta E_{oi}$	$\Delta\Delta E_{oi}$ a_1	$\Delta\Delta E_{oi}$ e
**Me_mH_{3-m}–CH**									
H_3–CH	0	–387.0	–139.2	0.0	–253.4	5.6			
Me_3–CH	3	–494.1	–175.4	–0.4	–334.2	15.9	–107.1	–36.2	–80.8
**Me_mH_{3-m}–$C^{\bullet}$**									
H_3–$C^{\bullet}$	0	–338.6	–98.8	0.0	–243.1	3.3			
Me_3–$C^{\bullet}$	3	–445.8	–137.8	–0.6	–325.5	18.0	–107.2	–39.0	–82.4

[a] Computed at M06-2X/TZ2P and, for each m, at equal substituent–carbon distances based on the geometry of $Me_mH_{3-m}C$–H. For H_3–$C^{\bullet}$: D_{3h} irreps merge to C_{3v} ones as: a_1’ + a_2” = a_1, a_2’ + a_1” = a_2 and e’ + e” = e. With ΔE_{oi} correction term for hybrid functional.

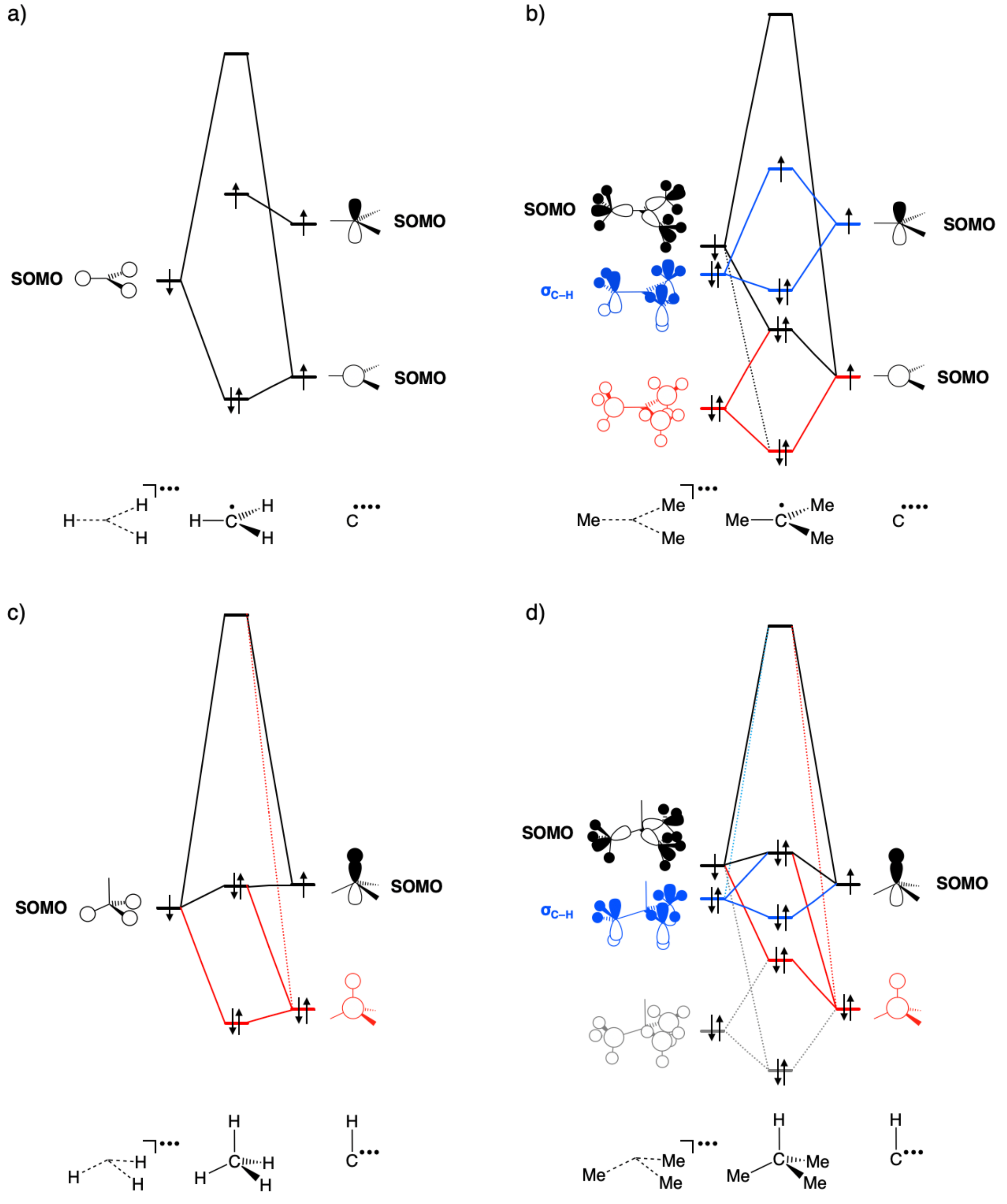

Figure S6. MO diagram in A_1' and A_2'' symmetry for a) $H_3C^{\bullet}$, and in A_1 symmetry for b) $Me_3C^{\bullet}$, c) H_3CH and d) Me_3CH in terms of $R_3^{\bullet\bullet\bullet}$ interacting with $C^{\bullet\bullet\bullet\bullet}$ or $CH^{\bullet\bullet\bullet}$. Interactions: 2c–2e⁻ in black, 2c–3e⁻ in red, 2c–3e⁻ hyperconjugation between $Me_3^{\bullet\bullet\bullet}$ σ_{C-H} and $C^{\bullet\bullet\bullet\bullet}$ p SOMO or $CH^{\bullet\bullet\bullet}$ p-type SOMO in blue. Computed at M06-2X/TZ2P at the equilibrium geometries.

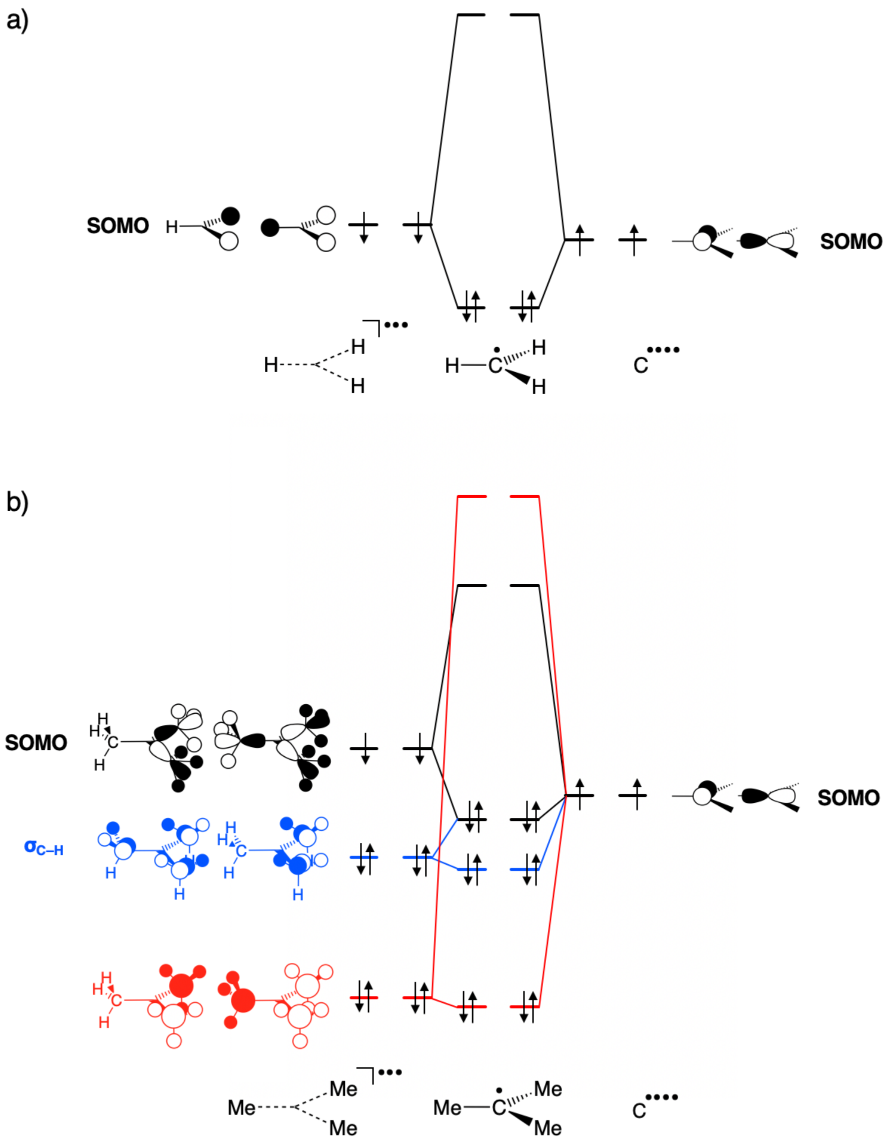

Figure S7. MO diagram in E symmetry for a) $H_3C^•$ and b) $Me_3C^•$. Interactions: 2c–2e⁻ in black, 2c–3e⁻ in red, 2c–3e⁻ hyperconjugation between $Me_3^{•••}$ $σ_{C–H}$ and the $C^{••••}$ or $CH^{•••}$ p SOMO in blue. Computed at M06-2X/TZ2P at the equilibrium geometries.

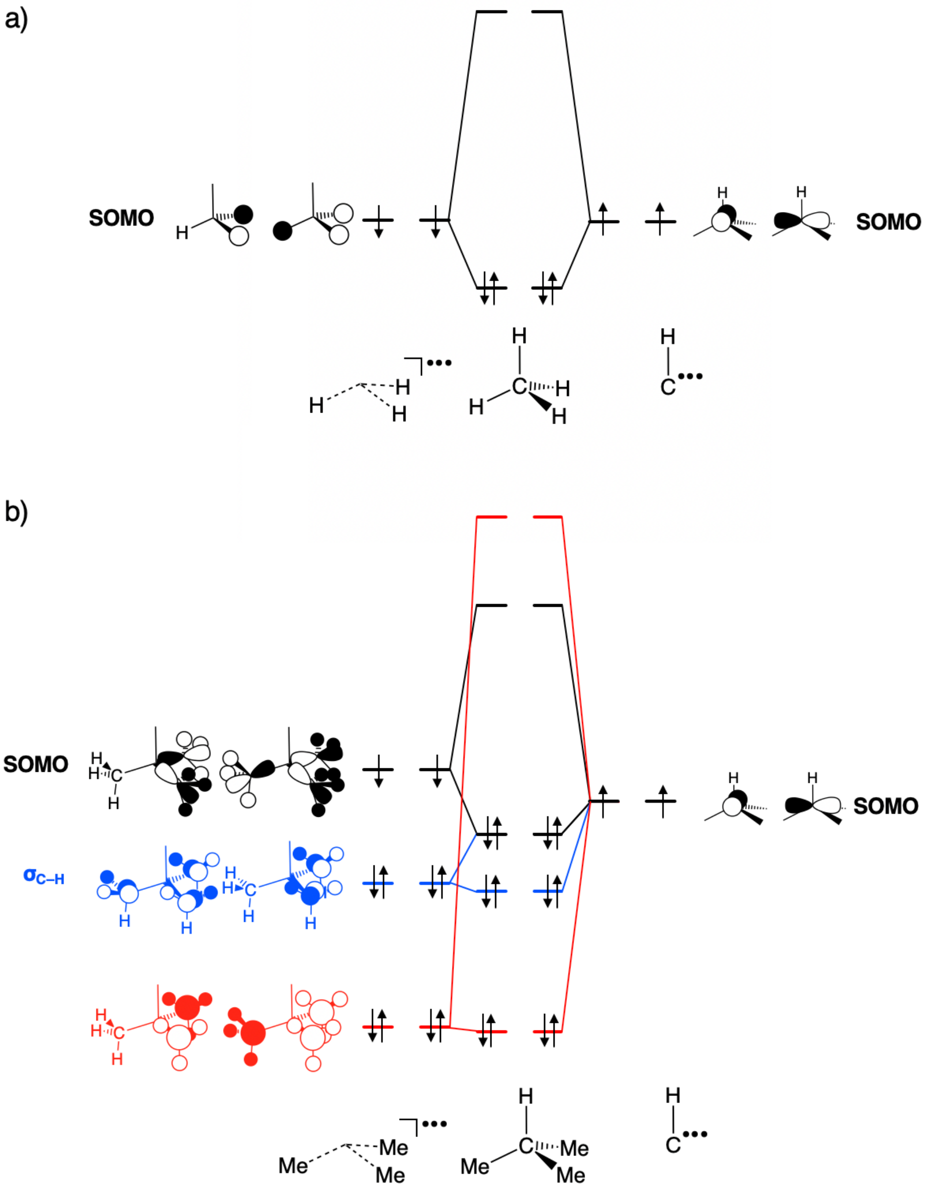

Figure S8. MO diagram in E symmetry for a) H_3CH and b) Me_3CH. Interactions: 2c–2e$^-$ in black, 2c–3e$^-$ in red, 2c–3e$^-$ hyperconjugation between $Me_3^{\bullet\bullet\bullet}$ $\sigma_{C–H}$ and $C^{\bullet\bullet\bullet\bullet}$ or $CH^{\bullet\bullet\bullet}$ p SOMO in blue. Computed at M06-2X/TZ2P at the equilibrium geometries.

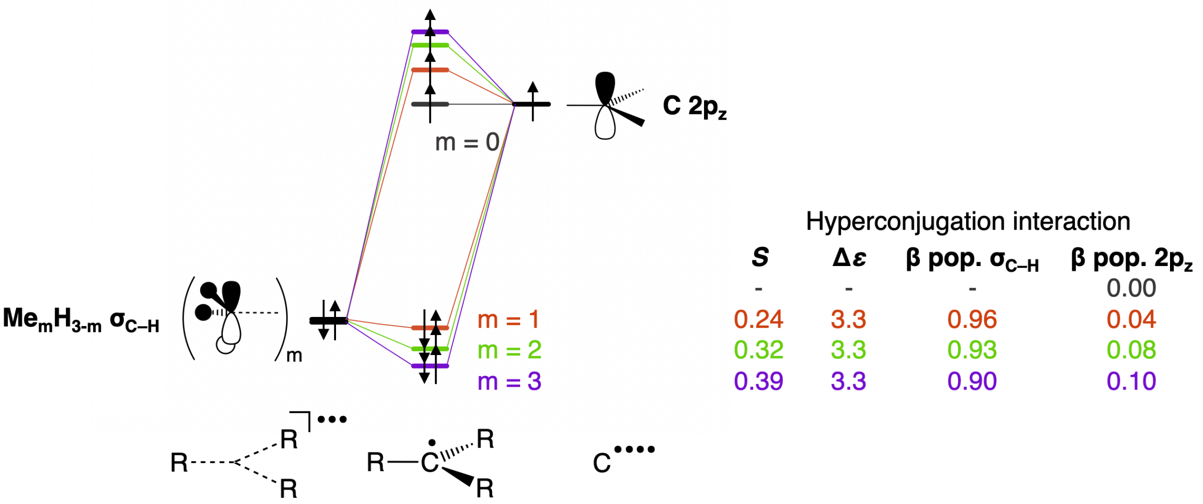

Figure S9. Schematic MO diagram for $Me_mH_{3-m}C^{\bullet}$ of the 2c–3e⁻ hyperconjugation between $Me_mH_{3-m}^{\bullet\bullet\bullet}$ σ_{C-H} and $C^{\bullet\bullet\bullet\bullet}$ p SOMO, where m = 0-3. Computed at M06-2X/TZ2P at the equilibrium geometries.

Table S9. Overlap S and energy gap Δε (in eV) of the 2c–2e⁻ interactions in $Me_mH_{3-m}CX$ and in $Me_mH_{3-m}C^{\bullet}$ in terms of $Me_mH_{3-m}^{\bullet\bullet\bullet}$ interacting with $CX^{\bullet\bullet\bullet}$ or $C^{\bullet\bullet\bullet\bullet}$, where m = 0-3 and X = H, OH.[a]

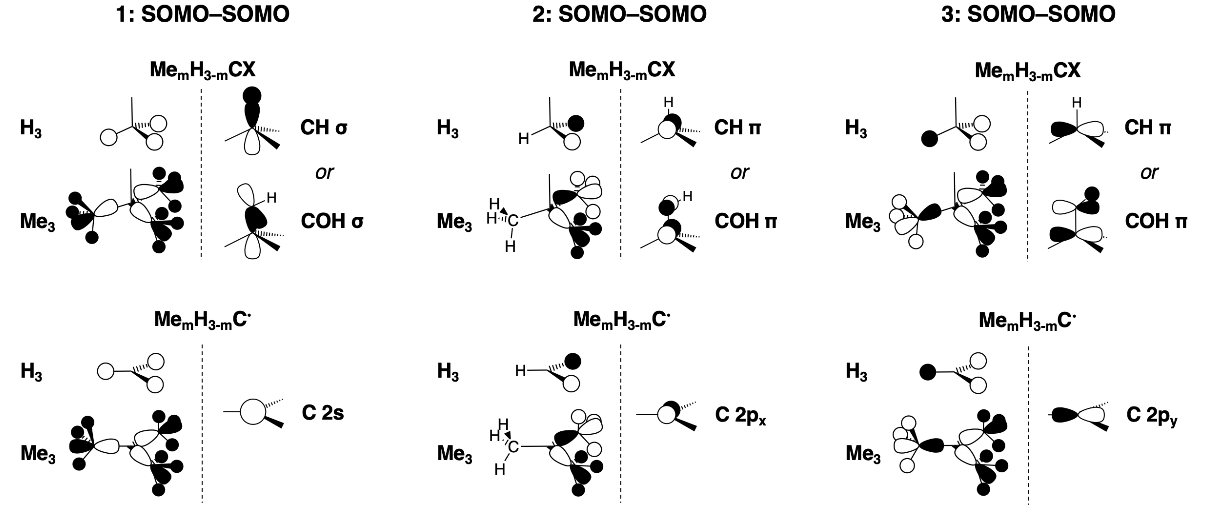

2c–2e⁻ interaction	1: SOMO–SOMO		2: SOMO–SOMO		3: SOMO–SOMO	
**System**	S	Δε	S	Δε	S	Δε
**H_3–CH**	0.55	1.5	0.74	0.1	0.74	0.1
**Me_3–CH**	0.33	1.2	0.45	1.7	0.45	1.7
**H_3–COH**	0.66	0.3	0.69	1.2	0.70	0.5
**Me_3–COH**	0.44	2.4	0.39	0.6	0.41	1.3
**H_3–$C^{\bullet}$**	0.83	6.3	0.76	0.8	0.76	0.8
**Me_3–$C^{\bullet}$**	0.65	8.5	0.47	2.6	0.47	2.6

[a] Computed at M06-2X/TZ2P at the equilibrium geometries. Fragment MOs are shown in the Table figure.

Table S10. Overlap S and energy gap Δε (in eV) of the 2c–3e⁻ hyperconjugation in $Me_mH_{3-m}CX$ and in $Me_mH_{3-m}C^•$ between $Me_mH_{3-m}^{•••}$ $σ_{C–H}$ and $CX^{•••}$ or $C^{••••}$ p SOMO, where X = H, OH and m = 0, 3.[a]

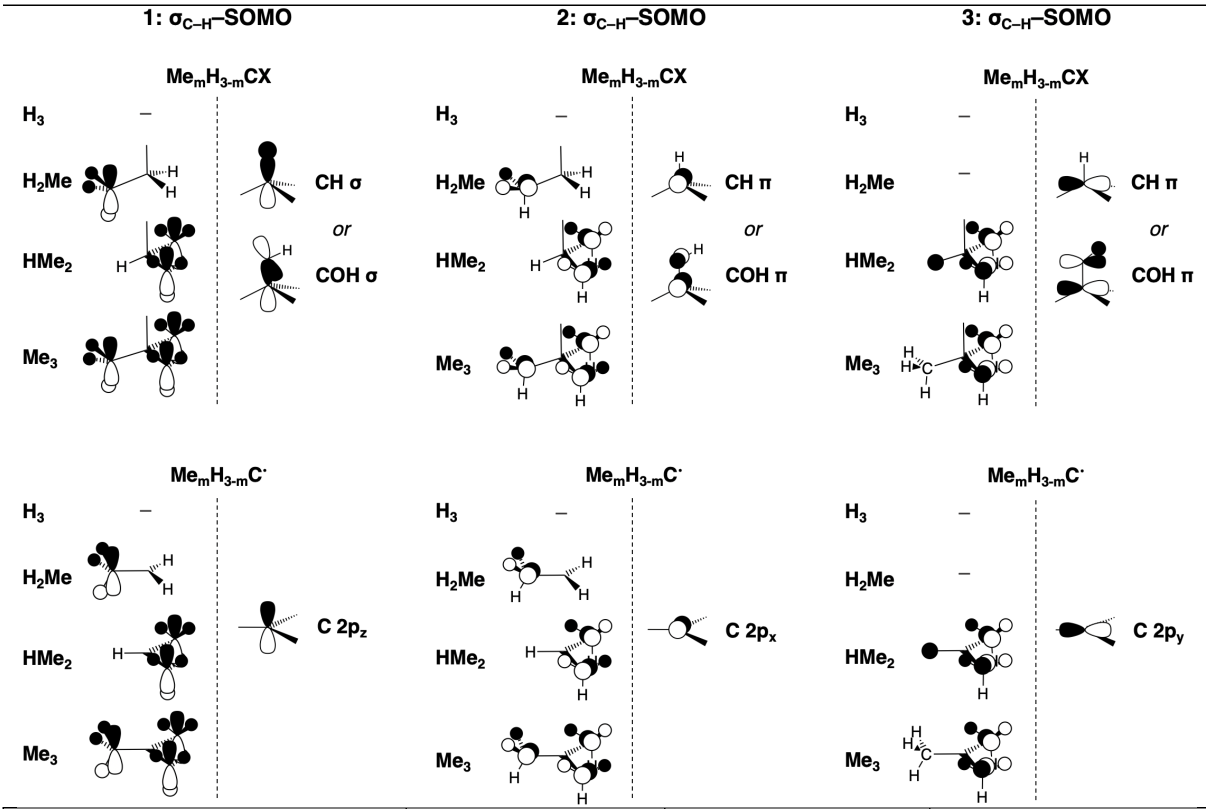

Hyperconjugation interaction	1: $σ_{C–H}$–SOMO		2: $σ_{C–H}$–SOMO		3: $σ_{C–H}$–SOMO	
**System**	S	Δε	S	Δε	S	Δε
**H_3–CH**	–	–	–	–	–	–
**H_2Me–CH**	0.18	0.9	0.27	4.4	–	–
**HMe_2–CH**	0.21	0.9	0.17	3.7	0.25	4.6
**Me_3–CH**	0.24	1.0	0.24	4.3	0.24	4.3
**H_3–COH**	–	–	–	–	–	–
**H_2Me–COH**	0.13	0.2	0.27	5.7	–	–
**HMe_2–COH**	0.10	0.3	0.14	4.2	0.24	5.9
**Me_3–COH**	0.15	0.1	0.24	5.5	0.23	4.8
**H_3–$C^•$**	–	–	–	–	–	–
**H_2Me–$C^•$**	0.24	3.3	0.28	3.5	–	–
**HMe_2–$C^•$**	0.32	3.3	0.16	2.9	0.30	3.6
**Me_3–$C^•$**	0.39	3.3	0.26	3.4	0.26	3.4

[a] Computed at M06-2X/TZ2P at the equilibrium geometries. Fragment MOs are shown in the Table figure.

Table S11. Overlap S and energy gap Δε (in eV) of 2c–3e⁻ interactions in $Me_mH_{3-m}CX$ or $Me_mH_{3-m}C^{•}$ in terms of $Me_mH_{3-m}^{•••}$ interacting with $CX^{•••}$ or $C^{••••}$, where X = H, OH and m = 0, 3.[a]

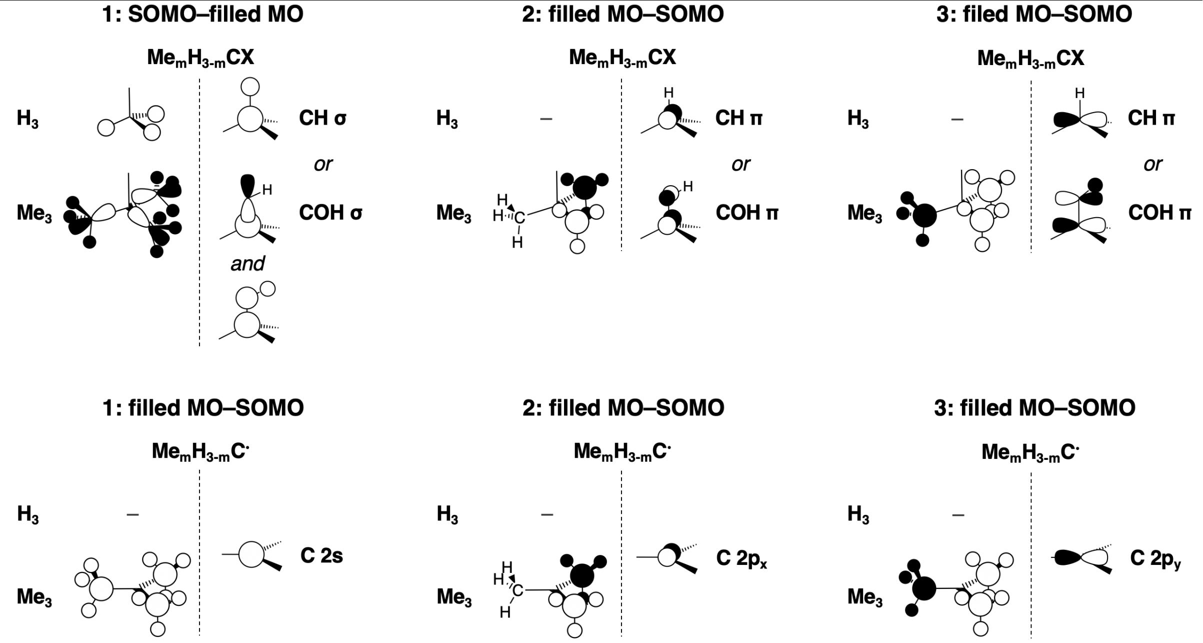

2c–3e⁻ interaction	1: SOMO–filled MO		2: filled MO–SOMO		3: filled MO–SOMO	
**System**	S	Δε	S	Δε	S	Δε
**H_3–CH**	0.64	6.7	–	–	–	–
**Me_3–CH**	0.53	9.4	0.46	12.1	0.46	12.1
**H_3–COH**	0.39	6.5	–	–	–	–
**Me_3–COH** with O 2p	0.32	9.2	0.46	12.6	0.46	13.3
**Me_3–COH** with O 2s	0.22	21.6				
	**1: filled MO–SOMO**		**2: filled MO–SOMO**		**3: filled MO–SOMO**	
	S	Δε	S	Δε	S	Δε
**H_3–$C^{•}$**	–	–	–	–	–	–
**Me_3–$C^{•}$**	0.47	2.1	0.48	11.1	0.48	11.1

[a] Computed at M06-2X/TZ2P at the equilibrium geometries. Fragment MOs are shown in the Table figure.

Table S12. Overlap S and energy gap Δε (in eV) of 2c–4e$^-$ interactions in Me_3CX in terms of $Me_3^{\bullet\bullet\bullet}$ interacting with $CX^{\bullet\bullet\bullet}$, where X = H, OH.[a]

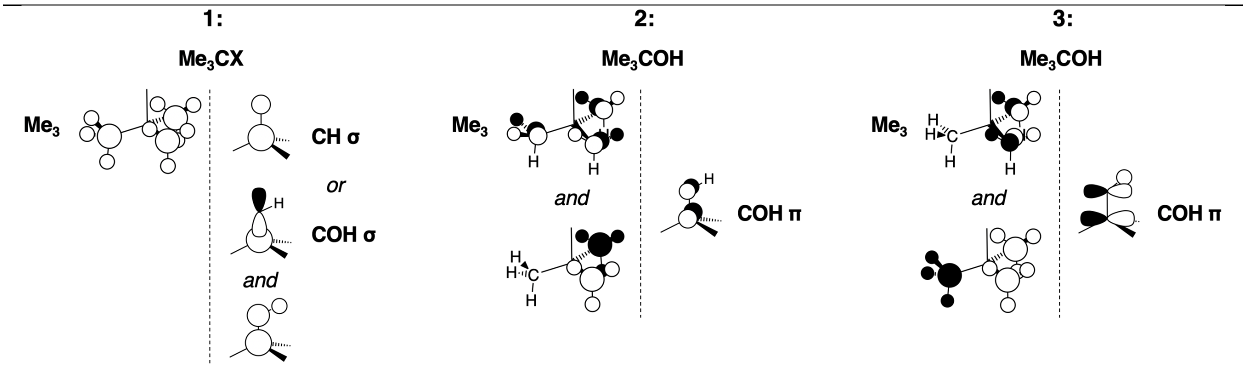

2c–4e$^-$ interaction<br>**System**	**1:**<br>S	**2:**<br>S	**3:**<br>S
**Me_3–CH**	0.33	–	–
**Me_3–COH**	0.21	0.09	0.05
**Me_3–COH**	0.10	0.14	0.10

[a] Computed at M06-2X/TZ2P at the equilibrium geometries. Fragment MOs are shown in the Table figure.

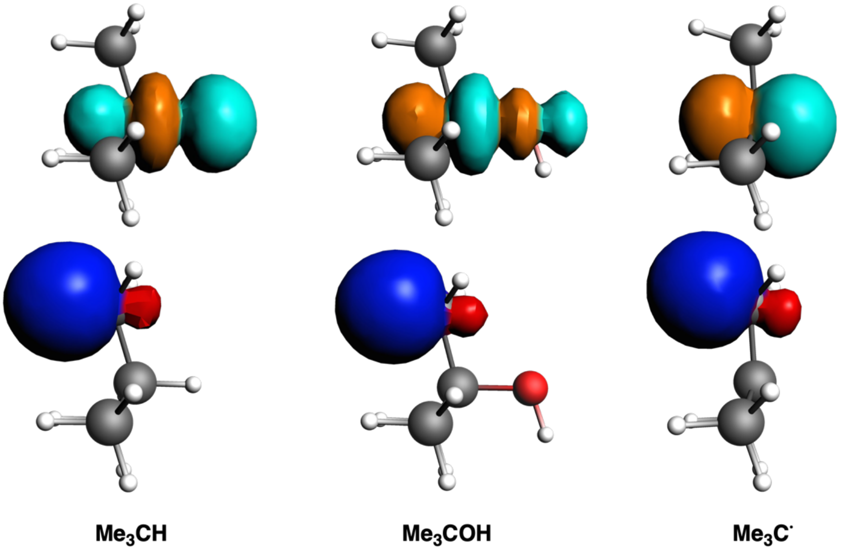

Figure S10. NBO orbitals involved in the hyperconjugation interaction between σ_{C-H} (bottom) and σ^*_{C-H}, σ^*_{C-O} or $2p_z$ SOMO (top) in Me_3CH, Me_3COH and $Me_3C\cdot$, respectively. Computed at M06-2X/cc-pVTZ//M06-2X/TZ2P level at the equilibrium geometries by means of NBO 6.0.[1]

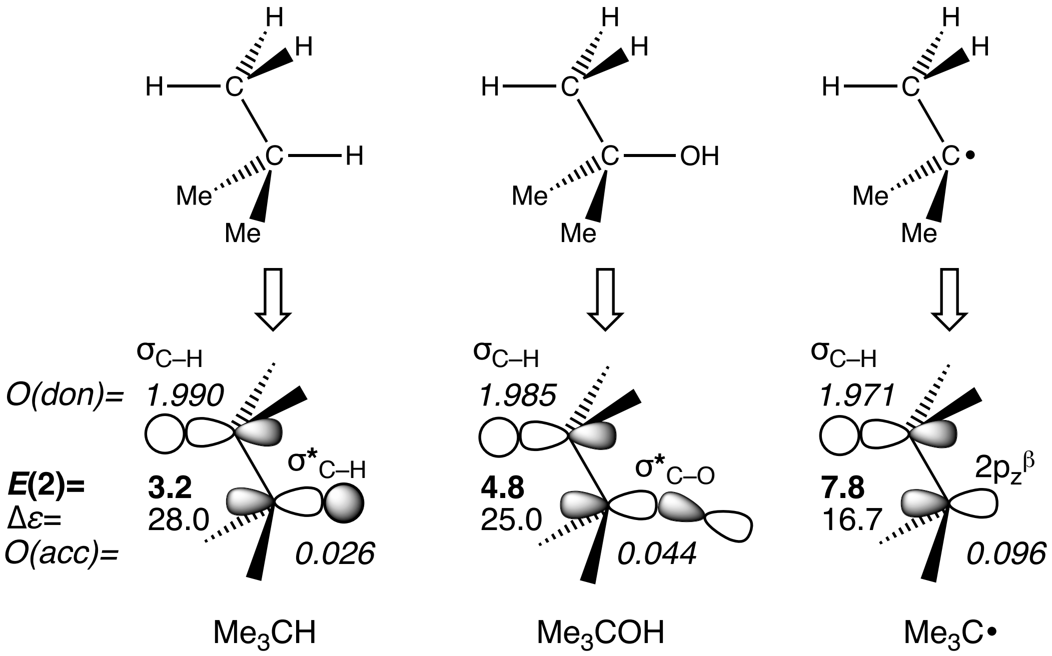

Figure S11. Schematic NBO orbital interaction diagram for Me_3CH, Me_3COH and $Me3C^{\bullet}$ of the hyperconjugation between σ_{C-H} and σ^*_{C-H}, σ^*_{C-O}, or $2p_z$ SOMO. Second-order perturbative estimates of the interactions in the NBO basis *E*(2) (in kcal/mol), occupancies of the donor and acceptor NBOs O(don) and O(acc) (in a.u.), and NBO energy gaps Δε (in eV) are enclosed. Computed at M06-2X/cc-pVTZ//M06-2X/TZ2P level at the equilibrium geometries by means of NBO 6.0.[1]

[1] Note that during the formation of NBOs some orbital interactions are lost that are visible for the canonical MOs, such as the 2c–3e$^-$ interactions that are depicted in Figure 4d for Me_3CH.

Table S13. Cartesian coordinates (in Å), ADF total energies [in brackets] (in kcal mol[−1]), and number of imaginary frequencies NIMAG of the equilibrium geometries of all closed-shell species in this study, computed at M06-2X/TZ2P.

**CH_4**	[-734.32]	NIMAG = 0	
H	0.627469	0.627469	-0.627469
C	0.000000	0.000000	0.000000
H	0.627469	-0.627469	0.627469
H	-0.627469	0.627469	0.627469
H	-0.627469	-0.627469	-0.627469

**CH_3CH_3**	[-1239.61]	NIMAG = 0	
C	0.000000	0.000000	0.762297
H	0.000000	1.015501	1.156540
H	0.879450	-0.507751	1.156540
H	-0.879450	-0.507751	1.156540
C	0.000000	0.000000	-0.762297
H	-0.879450	0.507751	-1.156540
H	0.000000	-1.015501	-1.156540
H	0.879450	0.507751	-1.156540

**$(CH_3)_2CH_2$**	[-1746.83]	NIMAG = 0	
H	0.873753	0.000000	-1.277079
H	0.880163	-1.296586	0.872428
C	0.000000	0.000000	-0.623032
H	-0.873753	0.000000	-1.277079
C	0.000000	-1.264220	0.228701
C	0.000000	1.264220	0.228701
H	0.880163	1.296586	0.872428
H	-0.880163	1.296586	0.872428
H	0.000000	2.164304	-0.384961
H	0.000000	-2.164304	-0.384961
H	-0.880163	-1.296586	0.872428

**$(CH_3)_3CH$**	[-2255.35]	NIMAG = 0	
H	0.000000	0.000000	1.506884
H	1.983239	-0.882944	0.289255
C	0.000000	0.000000	0.413547
C	1.449392	0.000000	-0.063417
C	-0.724696	-1.255210	-0.063417
C	-0.724696	1.255210	-0.063417
H	-1.756272	1.276063	0.289255
H	-0.743807	1.288311	-1.155237
H	-0.226967	2.159007	0.289255
H	-0.226967	-2.159007	0.289255
H	-0.743807	-1.288311	-1.155237
H	-1.756272	-1.276063	0.289255
H	1.983239	0.882944	0.289255
H	1.487613	0.000000	-1.155237

**$(CH_3)_4C$**	[-2764.49]	NIMAG = 0	
C	1.153164	-0.117674	0.045318
H	1.529785	0.897911	-0.088520
C	-0.375559	-0.140676	0.014459
C	-0.921349	0.738274	1.140525
C	-0.866410	0.392645	-1.332050
C	-0.867531	-1.576090	0.204142
H	-0.490763	-2.224419	-0.588879

H	-1.957937	-1.619275	0.186136
H	-0.529319	-1.980139	1.159932
H	-0.527612	1.417826	-1.491372
H	-1.956792	0.386217	-1.378999
H	-0.489459	-0.219045	-2.153578
H	-0.584062	0.377657	2.113849
H	-2.012761	0.738146	1.140025
H	-0.583811	1.769973	1.027226
H	1.528747	-0.494230	0.998373
H	1.567755	-0.739127	-0.750336

CH_3OH [-993.48] NIMAG = 0

H	1.444578	0.665135	0.000000
O	1.066915	-0.216340	0.000000
H	-0.716636	0.397993	0.889396
C	-0.343738	-0.115147	0.000000
H	-0.716636	0.397993	-0.889396
H	-0.734484	-1.129632	0.000000

CH_3CH_2OH [-1503.57] NIMAG = 0

O	1.426408	0.460926	0.000000
H	2.244731	-0.039621	0.000000
C	0.326462	-0.436362	0.000000
H	0.357531	-1.079678	-0.884409
H	0.357531	-1.079678	0.884409
C	-0.941958	0.387412	0.000000
H	-0.976540	1.023619	0.882920
H	-1.817625	-0.260237	0.000000
H	-0.976540	1.023619	-0.882920

$(CH_3)_2CHOH$ [-2014.26] NIMAG = 0

O	1.006370	-0.292831	0.106920
H	1.452095	0.537215	-0.079159
C	-0.396699	-0.110090	-0.045289
H	-0.753931	0.592738	0.715808
C	-0.727695	0.438815	-1.424074
C	-1.034417	-1.463513	0.196709
H	-0.684900	-2.174236	-0.552605
H	-2.119544	-1.392388	0.134192
H	-0.762382	-1.839621	1.181143
H	-0.248369	1.405715	-1.585759
H	-1.803145	0.574303	-1.538651
H	-0.377468	-0.252342	-2.191218

$(CH_3)_3COH$ [-2524.78] NIMAG = 0

O	-0.036444	1.666716	0.000000
H	0.874908	1.972698	0.000000
C	-0.040064	0.238405	0.000000
C	0.658089	-0.276050	-1.255039
C	0.658089	-0.276050	1.255039
C	-1.508329	-0.157401	0.000000
H	-2.002791	0.243972	0.883746
H	-1.612233	-1.241977	0.000000
H	-2.002791	0.243972	-0.883746
H	1.704872	0.034805	1.265792
H	0.630333	-1.365065	1.297979
H	0.170578	0.123117	2.143690
H	0.170578	0.123117	-2.143690

H	0.630333	-1.365065	-1.297979
H	1.704872	0.034805	-1.265792

Table S14. Cartesian coordinates (in Å), ADF total energies [in brackets] (in kcal mol^{-1}), and number of imaginary frequencies NIMAG of the equilibrium geometries of all radical species in this study, computed at M06-2X/TZ2P.

H• [-51.65] NIMAG = 0

CH_3• [-571.09] NIMAG = 0

C	0.000000	0.000000	0.000000
H	0.538117	0.932046	0.000000
H	0.538117	-0.932046	0.000000
H	-1.076234	0.000000	0.000000

CH_3CH_2• [-1080.29] NIMAG = 0

C	0.035708	0.863377	0.000000
H	-0.041407	1.415891	0.923194
H	-0.041407	1.415891	-0.923194
C	0.021404	-0.621667	0.000000
H	0.513780	-1.027896	-0.883455
H	-1.001859	-1.017701	0.000000
H	0.513780	-1.027896	0.883455

$(CH_3)_2CH$• [-1590.47] NIMAG = 0

C	0.058376	0.615201	0.000000
H	-0.136411	1.677746	0.000000
C	0.021140	-0.128029	-1.287151
C	0.021140	-0.128029	1.287151
H	0.760543	-0.933602	1.297425
H	-0.954386	-0.605291	1.450577
H	0.211721	0.520448	2.140391
H	0.211721	0.520448	-2.140391
H	-0.954386	-0.605291	-1.450577
H	0.760543	-0.933602	-1.297425

$(CH_3)_3C$• [-2101.05] NIMAG = 0

H	1.737422	-1.239113	0.466435
H	-1.941815	0.885095	0.466435
H	-1.941815	-0.885095	0.466435
H	-1.736422	0.000000	-1.039293
C	0.000000	0.000000	0.230055
C	-1.477230	0.000000	0.029737
C	0.738615	-1.279319	0.029737
C	0.738615	1.279319	0.029737
H	0.204393	2.124208	0.466435
H	0.868211	1.503785	-1.039293
H	1.737422	1.239113	0.466435
H	0.868211	-1.503785	-1.039293
H	0.204393	-2.124208	0.466435

OH• [-323.14] NIMAG = 0

H	1.444227	0.705040	0.000000
O	1.090100	-0.200647	0.000000

CH••• [-227.81] NIMAG = 0

H	0.000000	0.000000	-1.372732
C	0.000000	0.000000	-0.281844

CCH_3••• [-740.07] NIMAG = 0

C	2.018292	1.037834	0.000000
C	1.301626	-0.267040	0.000000
H	1.750736	1.615527	0.887863
H	1.750736	1.615527	-0.887863
H	3.098677	0.875370	0.000000

COH••• [-481.27] NIMAG = 0

O	1.099373	-0.169410	0.000000
H	1.331971	0.769055	0.000000
C	-0.250056	-0.293279	0.000000
